# Dietary Flaxseed and Flaxseed Oil Differentially Modulate Aspects of the Microbiota Gut–Brain Axis Following an Acute Lipopolysaccharide Challenge in Male C57Bl/6 Mice

**DOI:** 10.3390/nu15163542

**Published:** 2023-08-11

**Authors:** Dawson B. H. Livingston, Allison Sweet, Alexane Rodrigue, Lalit Kishore, Julia Loftus, Farida Ghali, Salma Mahmoodianfard, Colleen Celton, Farah Hosseinian, Krista A. Power

**Affiliations:** 1 Faculty of Medicine, Department of Biochemistry, Microbiology and Immunology, University of Ottawa, Ottawa, ON K1H 8L1, Canada; dlivi057@uottawa.ca (D.B.H.L.); aswee059@uottawa.ca (A.S.); arodr038@uottawa.ca (A.R.); 2 Faculty of Health Science, School of Nutrition Sciences, University of Ottawa, Ottawa, ON K1N 6N5, Canada; lkishore@uottawa.ca (L.K.); fghal087@uottawa.ca (F.G.); 3 Faculty of Science, Department of Biochemistry, University of Ottawa, Ottawa, ON K1N 6N5, Canada; jloft040@uottawa.ca; 4 Faculty of Health Science, School of Human Kinetics, University of Ottawa, Ottawa, ON K1N 6N5, Canada; smahm005@uottawa.ca; 5 Faculty of Science, Department of Chemistry, Carleton University, Ottawa, ON K1S 5B6, Canada; colleencelton@cmail.carleton.ca (C.C.); farah.hosseinian@carleton.ca (F.H.); 6 Faculty of Science, Institute of Biochemistry, Carleton University, Ottawa, ON K1S 5B6, Canada

**Keywords:** LPS, lipopolysaccharide, gut–brain axis, microbiota, microbiota gut–brain axis, Qiime2, PICRUSt2, flaxseed, flaxseed oil, inflammation

## Abstract

The microbiota gut–brain axis (mGBA) is an important contributor to mental health and neurological and mood disorders. Lipopolysaccharides (LPS) are endotoxins that are components of Gram-negative bacteria cell walls and have been widely shown to induce both systemic and neuro-inflammation. Flaxseed (*Linum usitatissimum*) is an oilseed rich in fibre, n3-poly-unsaturated fatty acid (alpha-linolenic acid (ALA)), and lignan, secoisolariciresinol diglucoside, which all can induce beneficial effects across varying aspects of the mGBA. The objective of this study was to determine the potential for dietary supplementation with flaxseed or flaxseed oil to attenuate LPS-induced inflammation through modulation of the mGBA. In this study, 72 5-week-old male C57Bl/6 mice were fed one of three isocaloric diets for 3 weeks: (1) AIN-93G basal diet (BD), (2) BD + 10% flaxseed (FS), or (3) BD + 4% FS oil (FO). Mice were then injected with LPS (1 mg/kg i.p) or saline (*n* = 12/group) and samples were collected 24 h post-injection. Dietary supplementation with FS, but not FO, partially attenuated LPS-induced systemic (serum TNF-α and IL-10) and neuro-inflammation (hippocampal and/or medial prefrontal cortex IL-10, TNF-α, IL-1β mRNA expression), but had no effect on sickness and nest-building behaviours. FS-fed mice had enhanced fecal microbial diversity with increased relative abundance of beneficial microbial groups (i.e., Lachnospiraceae, *Bifidobacterium*, Coriobacteriaceae), reduced *Akkermansia muciniphila*, and increased production of short-chain fatty acids (SCFAs), which may play a role in its anti-inflammatory response. Overall, this study highlights the potential for flaxseed to attenuate LPS-induced inflammation, in part through modulation of the intestinal microbiota, an effect which may not be solely driven by its ALA-rich oil component.

## 1. Introduction

The microbiota gut–brain axis (mGBA) is now recognized as an important contributor to a number of mental health conditions including neurodegenerative and mood disorders [1,2,3]. A dysbiotic intestinal microbial community [4,5] and a dysfunctional intestinal barrier [6,7,8] are two factors shown to be altered in several mental health conditions and, thus, may be potential targets for therapeutic intervention. This may be due, in part, to chronic low-grade inflammation driven by enhanced exposure to bacterial components and metabolites. Peripheral and central exposure to lipopolysaccharide (LPS), a pathogen-associated molecular pattern (PAMP) expressing component of the cell wall of Gram-negative bacteria, results in innate immune responses (e.g., secretion of proinflammatory cytokines interleukin (IL)-1β, IL-6, IL-10, and tumour necrosis factor-α (TNF-α)) [9,10] driven through Toll-like receptor 4 (TLR-4)-dependent and -independent cell signalling cascades [11]. The immune responses are mediated by dendritic cells, macrophages, endothelial and epithelial cells [12,13], and cells of the central nervous system (CNS) including microglia, astrocytes, and neurons [14,15,16]. In rodent models, LPS-induced neuroinflammation, especially in the medial prefrontal cortex (mPFC) and hippocampus (HIP), results in impaired cognitive function [17,18], dysregulation of neurogenesis [19,20], and behaviours indicative of anxiety and depression [21,22,23,24,25]. Therefore, therapies that can modulate the mGBA may play an important role in attenuating LPS-induced inflammation and improving mental health conditions. 

The composition and function of the intestinal microbiota may be a potential modulator of the LPS-induced inflammatory response. Intraperitoneal (i.p) LPS exposure has been shown to alter the intestinal microbiota composition and/or decrease microbial diversity in mice [26,27,28] and piglets [29], which may potentiate the peripheral and central inflammatory LPS responses. LPS treatment (ex vivo) of liver samples harvested from either germ-free (GF) or specific-pathogen-free (SPF) mice, showed that samples from GF mice were more susceptible to LPS-induced inflammation [30], suggesting that the microbiome may have important roles in mediating inflammation. Furthermore, mice consuming a fermentable fiber-rich diet displayed an attenuated immune response in microglia following LPS exposure, compared to mice consuming a fiber-poor diet [31]. This beneficial response was shown to be due to the anti-inflammatory effects of the microbial-derived short-chain fatty acids (SCFAs) (e.g., acetate and butyrate), demonstrating that diet-induced improvements in the function of the intestinal microbial community can help mitigate neuroinflammation. Of similar relevance, probiotic supplementation in mice has been shown to attenuate LPS-induced intestinal inflammation and microbial dysbiosis [28], suggesting that improving the microbiota can attenuate the LPS response. These preclinical findings are also supported by observational studies, which show that populations who consume fiber-rich diets (e.g., plant-based or Mediterranean diets) [32,33], or alter their microbiota through probiotics [34], also display reduced inflammation and mental health disorders.

Flaxseed (*Linum usitatissimum*) is a relatively inexpensive and accessible dietary component that may beneficially target the mGBA to improve mental health. Flaxseed is an oilseed that is rich in fermentable dietary fibres, which, when metabolized by the microbiota, produces anti-inflammatory SCFAs in both humans and in rodents [35,36]. Flaxseed in also rich in the n3-polyunsaturated fatty acid (PUFA), alpha-linolenic acid (ALA), which can be metabolized to the long-chain n3-PUFAs, eicosapentaenoic acid (EPA), and docosahexaenoic acid (DHA), which have been shown collectively to attenuate LPS-induced inflammation in rodent models [21,22,24,25,37,38]. Previous studies have shown the potential for dietary supplementation with flaxseed and/or flaxseed oil to alter the composition and/function of the intestinal microbiota in rodent models [36,39,40,41], however, the downstream implications of these changes, with a focus on the mGBA, has not been investigated. Furthermore, a daily gavage with FS oil (FO) for 2 weeks resulted in decreased systemic mediators of inflammation and anxiety-like behavior in a rodent model of post-partum depression [42]. Flaxseed is a rich source of the lignan, secoisolariciresinol diglucoside (SDG), which, when metabolized by the intestinal microbiota, generates microbial metabolites, enterodiol (ED), and enterolactone (EL), which have anti-inflammatory and antioxidant properties [43,44]. Further, SDG has been shown to attenuate LPS-induced blood–brain barrier (BBB) permeability and reduce neuroinflammation in mice [45]. Thus, due to its diverse profile of beneficial components, flaxseed may serve as a dietary intervention for improved mental health through modulation of the mGBA.

Therefore, the objective of this study was to determine the therapeutic potential of flaxseed and one of its bioactive components, flaxseed oil, on LPS-induced inflammation, through modulation of the mGBA. We hypothesized firstly that mice receiving an i.p LPS injection would exhibit negative changes across the mGBA, highlighted by microbial dysbiosis, and systemic and neuro-inflammation; secondly, mice fed diets supplemented with flaxseed or flaxseed oil would attenuate the response to LPS through modulation of the gut microbiota, and the systemic/central immune response; and finally, compared to mice consuming flaxseed oil alone, mice consuming diets supplemented with flaxseed would further attenuate the LPS-induced effects due to the combined beneficial effects of dietary fibre, lignan, and ALA. 

## 2. Materials and Methods

### 2.1. Flaxseed Nutrient Composition, Oil Extraction, and Oil Fatty Acid Composition

Brown flaxseeds (*Linum usitatissimum*) were generously donated by Natunola (Winchester, ON, Canada). Whole ground flaxseeds were sent to Bureau Veritas (Mississauga, ON, Canada) for proximate analysis (fat, lipid, carbohydrate), and dietary fibre (soluble and insoluble) content (Table S1). Flaxseed oil was extracted from whole flaxseeds by Dr. Hosseinian’s laboratory at Carleton University using an automatic oil cold press (Huanyu, CZR 309 110V). The resulting oil was filtered and was left at −4 °C to winterize overnight to remove impurities. The supernatant was collected and centrifuged at 1500 RPM for 15 min and stored at 4 °C until use. The flaxseed oil fatty acid profile (Table S2) was analysed by the Lipid Analytical Laboratory (Guelph, ON, Canada; https://www.lipidanalytical.com/services/fatty-acid-profiling (accessed on 30 July 2023)) using the Modified AOCS Official Method—Ce-1i-07.

### 2.2. Experimental Diet Preparation

Three isocaloric diets were prepared by Envigo (Madison, WI, USA): (1) a modified basal diet (BD) (AIN-93G with 7% corn oil); (2) BD supplemented with 10% whole ground flaxseed (FS); and (3) BD supplemented with 4% flaxseed oil (FO). The amount of flaxseed oil in both the FS and FO diets were equivalent. The complete nutritional composition of the diets is shown in Table 1. The experimental diets were stored at −20 °C to prevent fatty acid oxidation. 

### 2.3. Animal and Experimental Design

C57Bl/6 male mice (5 weeks old; *n* = 72) were purchased from Charles River, (Kingston, NY, USA), and were individually caged with corn cob bedding and cotton nesting material and had access to BD and water ad libitum for a 1-week acclimatization period. The mice were maintained on a 12 h light:dark cycle, with room temperature at 23 °C, and humidity at 40%. All experimental conditions were reviewed and approved by the University of Ottawa Animal Care Committee (animal use protocol: #HSe-2857-R3).

**Figure 1:**
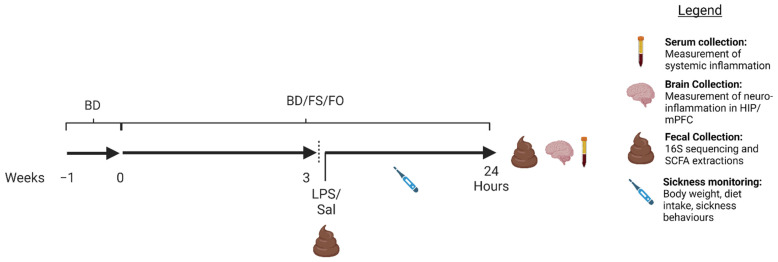
Experimental design. Seventy-two, 5-week-old male C57Bl/6 mice were acclimatized for 1 week on a BD, then randomized by body weight and given either a BD, FS, or FO diet for 3 weeks. The mice were again randomized by body weight within diet, fecal pellets were collected, and mice were given an i.p injection of LPS (1 mg/kg) or SAL. The mice were monitored for sickness behaviours for 24 h post-injection, then fecal pellets were collected, and the mice were euthanized. Serum and brain samples (hippocampus (HIP), medial prefrontal cortex (mPFC)) were collected. Figure created with Biorender.com. BD: basal diet. FS: flaxseed; FO: FS oil; LPS: Lipopolysaccharides; Sal: saline.

Following acclimatization, mice were randomized by body weight (BW) into three experimental diet groups (*n* = 24/group); (1) BD, (2) FS, and (3) FO. The mice were fed their respective diets for 3 weeks, during which BW and diet intake were measured three times a week using a digital scale. Diets were replaced three times a week with fresh diet, to prevent excessive oxidation of the fatty acids. After 3 weeks, feces were collected, and mice were given an intraperitoneal (i.p.) injection of LPS (1 mg/kg BW) (*n* = 12/group) or saline (SAL) (*n* = 12/group). The mice were monitored throughout the day to assess well-being (see Section 2.5). Twenty-four hours post-injection, feces were collected, and mice were euthanized by decapitation. Trunk blood was collected for serum isolation and tissues were collected and stored at −80 °C for later analyses (Figure 1).

### 2.4. LPS Preparation

LPS from Escherichia coli O26:B6 (Sigma, St. Louis, MO, USA; L3755-100MG) was dissolved in 0.9% sodium chloride saline (Fresenius Kabi Canada, Toronto, ON, Canada; 02395150), to create a 5 mg/mL stock solution. The stock solution was aliquoted into DNAse, RNAse-free microcentrifuge tubes, and stored at −80 °C until needed. On the day of injection, one aliquot was removed from the freezer and diluted to 0.2 mg/mL with the 0.9% sodium chloride saline.

### 2.5. Well-Being Measures Post-LPS/SAL Injection

#### 2.5.1. Diet Intake (DI) and Body Weight (BW)

BW and DI were measured 1 h prior to LPS/SAL injection, and again at 8 and 24 post-injection. DI was calculated by diet weight (pre-injection)–diet weight (8 and 24 h) post-injection. The percent BW change was calculated by (BW post-injection–BW pre-injection)/BW pre-injection × 100.

#### 2.5.2. Analysis of Sickness Behaviours

Sickness behaviours were recorded at 30 min, and 2, 4, 6, 8, and 24 h post-injection (adapted from [46]). The behaviours measured include piloerection (hair standing up), lethargy (slow-moving), ptosis (squinted eyes), and huddling (hunched back). The behaviours were scored based on the presence (1) or absence (0) by two blinded observers, and the average of the four behaviours was the overall sickness score (max score = 4). 

#### 2.5.3. Nest Quality Scoring

Immediately before injection of LPS or SAL, the used cotton nesting material was removed and discarded. Two new squares of cotton nesting material were added to the front of the cage, and videos of the nests were recorded 8- and 24-h post-injection. The videos were scored by two blinded observers using four key attributes adapted from [47,48]: (i) nesting material shredding (0–2.5), (ii) nest shape (0–1.5), (iii) nest location (0–1), and (iv) nest walls (0–1.5). The maximum score was 6.5. The scoring system and representative images of the nests are outlined in Table S3 and Figure S2.

### 2.6. Assessment of Systemic Inflammation

Immediately following blood collection at euthanasia, blood was kept at room temperature for 30 min to allow clotting, then put on ice before centrifugation. The blood was centrifuged for 10 min at 10,000× *g* at 4 °C, after which the serum was collected and separated into 50 μL aliquots and stored at −80 °C until use. Additionally, the spleen was excised and weighed to determine the relative spleen weight, which may be used as a marker of an increased immune response [49].

The serum concentrations of IL-1β, TNF-α, and IL-10 were measured by a custom Milliplex Mouse High Sensitivity T Cell Magnetic Bead Panel (Millipore, Burlington, MA, USA; MHSTCMAG-70K-03), as per the manufacturer’s instructions. Of note, the concentration of IL-1β in most samples were below detectable limit, and, thus, were excluded from this study. The resultant plate was then read by the Magpix Multiplex Reader (Luminex, Austin, TX, USA), and data were acquired using Luminex xPonent for Magpix (v4.2), then analyzed by Milliplex analyst (v5.1.0.0) (VigeneTech Inc., Carlisle, MA, USA) and presented as concentration (pg/mL). 

### 2.7. Hippocampus (HIP) and Medial Prefrontal Cortex (mPFC) Isolation and Gene Expression of Inflammatory Cytokines

After euthanasia, the brain was promptly isolated. With the brain on a chilled surface, the olfactory bulbs were removed and discarded. A ~1 mm section of the prefrontal cortex was excised, which was further dissected to excise the medial PFC by collecting the medial 2 mm (the inside of the white matter) and it was stored in a microcentrifuge tube on dry ice. To extract the HIP, the cerebellum was removed and stored, and the brain was then cut to separate the two hemispheres. With the medial surface facing up, the midbrain, striatum and thalamus were peeled back to reveal the HIP, which was then collected and stored in a microcentrifuge tube on dry ice [50]. This procedure was completed for both hemispheres of the brain, then tissues were stored at −80 °C until further analysis. 

RNA was extracted and purified from the HIP and mPFC using the Total RNA Purification Plus Kit (Norgen Biotek, Thorold, ON, Canada; #48400). The purified RNA (1 μg) was used to prepare complementary DNA (cDNA) using the High-Capacity cDNA Reverse Transcription Kit (Applied Biosystems, Waltham, MA, USA; #4368814). The relative expression of mRNA in the resultant cDNA was measured by quantitative PCR (qPCR) using SYBR green (Applied Biosystems, Waltham, MA, USA; #4367659) and the CFX96 Touch (Bio-rad, Hercules, CA, USA) real-time system. The relative gene expression was then calculated using the ΔΔCq method, using the housekeeping gene RPLP0 as a baseline. Primer sequences are shown in Table S4.

### 2.8. Fecal DNA Extraction and 16S rRNA Gene Sequencing

Fecal pellets (3–4) were collected from the mice immediately prior to injection, and at 24 h post-injection, by placing a mouse into a clean empty cage for a maximum of 5 min, then all pellets produced were transferred into microcentrifuge tubes on dry ice, and were stored at −80 °C until later use. Fecal DNA was extracted using QIAamp Fast DNA Stool Mini Kit (Qiagen, Hilden, Germany; #51604) with the recommended protocol. DNA concentration was determined using the Quant-iT PicoGreen dsDNA Assay kit (Thermo Fisher Scientific, Waltham, MA, USA; #P11496) as per the manufacturer’s instructions. The 16S rRNA library (V3–V4 regions) was prepared following Illumina guidelines. In brief, the 16S regions were amplified (primer sequences are shown in Table S4) by the following program: 95 °C for 30 s, 55 °C for 30 s), then 72 °C for 5 min. The PCR product was verified on a 1.5% agarose gel. The amplified 16S V3-V4 region was then purified using AMPure XP beads (Beckman Coulter Genomics, Brea, CA, USA; # A63881). Dual indices and Illumina sequencing adaptors were then added to the purified 16S V3–V4 regions using Nextera XT index primers (Illumina, San Diego, CA, USA; #FC-131-1001). The pooled samples were loaded into the Miseq reagent kit V3 600 cycle cartridge (Illumina, San Diego, CA, USA; MS-102-3003), and placed in the Illumina Miseq (Illumina, Miseq) for sequencing. 

#### 16S rRNA Data Processing and Bioinformatics Analyses

The resultant dual-index paired-end reads were analyzed using Qiime2 (version 2022.2) [51]; the sequences were subjected to quality control, specifically denoising and chimaera filtering using the Divisive Amplicon Denoising Algorithm 2 (DADA2) Qiime2 plugin [52]. Resultant features were aligned with MAFFT [53] and the phylogenetic tree was generated using FastTree analysis [54]. The resulting taxonomy was classified using GreenGenes classifier 13.8 and the sklearn naïve Bayes classifier [55,56,57]. Features were filtered to include only features with a minimum of 500 frequency and 5 samples. Alpha diversity metrics (Shannon’s diversity [58], Faith’s phylogenetic diversity [59], and observed features), and a beta diversity metric (Bray–Curtis [60]) were analyzed using the Qiime2 core-metric method at a sampling depth of 15,000. The dissimilarities among samples/communities (Bray–Curtis) were visualized by principal coordinate analysis (PCoA) using MicrobiomeAnalyst [61,62], and the axis text fonts and size were changed to improve visibility using Inkscape (V1.2.1, Inkscape developers). Significance between pre-injection alpha diversity metrics were measured by Kruskal–Wallis test with Benajmini–Hochberg corrections in Qiime2. Group significance between beta diversity indices (Bray–Curtis) were assessed by PERMANOVA [63] in Qiime2. Taxa bar plots of relative abundances were created for visualization in GraphPad Prism 9 (v9.4.0). The differences in relative abundance were assessed by Linear discriminant analysis effect size analysis (LEfSe) [64] through the Galaxy Server (https://huttenhower.sph.harvard.edu/galaxy/ (accessed on 5 July 2023)) with an LDA threshold of 2, and *p*-value cut-off of 0.05. The LEfSe results were also visualised using a cladogram, which depicts the significantly different taxa in a circular hierarchical tree.

The inferred function of the microbiota was analysed using phylogenetic investigation of communities by reconstruction of unobserved states 2 (PICRUSt2) [65,66]. First, the fragment insertion Qiime2 plugin [67] was used to place sequences on a reference phylogeny, after which the remainder of the PICRUST2 pipeline was subsequently run. Specifically, hidden-state predictions (hsp.py) were run to obtain the Kyoto Encyclopedia of Genes and Genomes (KEGG) orthologs [68] (KO) abundances per predicted genome. The metagenome_pipeline.py command was then run to adjust the KO abundances by 16S abundance. The resulting file (pred_metagenome_unstrat.tsv) included KO abundances per mouse. This file was used to determine the inferred function of the microbiota in RStudio (R = V4.2.1, R Studio: V2022.07.1+554). Specifically, using a custom R code analogous to the categorize_by_function.py from PICRUST1 [69], the KOs were mapped to a manually curated mapping file derived from https://www.genome.jp/kegg-bin/get_htext?ko00001.keg (accessed on 17 June 2022) [68]. The resulting functional outputs were visualized using Graphpad Prism and statistical analysis of taxonomic and functional profiles (STAMP) [70].

### 2.9. Fecal Short-Chain Fatty Acid Analyses 

The feces were weighed and placed into the FreeZone 12 Liter Console Freeze Dry System (Labconco, Kansas City, MO, USA; #7759030) for 18–20 h. The dried feces were mixed with MilliQ water (10 × dry weight) and vortexed thoroughly for 15 s. The samples were incubated at RT for 3 h, while vortexing every 30 min for 15 s. The samples were then centrifuged for 30 min at 14,000× *g* at 4 °C. The pH of the supernatant was recorded (Thermo Scientific, Waltham, MA, USA; Orion Star A111 pH Benchtop Meter, Thermo Scientific ROSS MICRO PH ELECTRODE), then 10 μL of internal standard (IS) (comprised of 5.5 mM 2-ethylbutyric acid (Sigma-Aldrich, St. Louis, MO, USA; #109959-100ML) in 100% formic acid (Sigma-Aldrich, St. Louis, MO, USA; #33015-1L)) was added to 100 μL of each sample (10% IS), after which, pH was recorded again. The samples were filtered using 13 mm syringe filters (0.20 μm pore size) (VWR, Mont-Royal, QC, Canada; #CA28145-491), then placed into glass inserts (Thermo Fisher Scientific, Waltham MA, USA; #C4010-630) and into 11mm amber glass Snap-it Target LoVials (Thermo Fisher Scientific, Waltham, MA, USA; #C4011-6W) with 11mm autosampler vial Snap-it Snap Caps (Thermo Fisher Scientific, Waltham, MA, USA; #C4011-50).

To quantify the SCFA concentrations, a standard curve was created for the gas chromatograph (GC) (Shimadzu, Kyoto, Japan; Nexis GC-2030) using a serially diluted Volatile Free Fatty Acid Mix (Millipore Sigma, Burlington, MA, USA; 46975-U) to make the following concentrations: 0.078 mM, 0.156 mM, 0.313 mM, 0.625 mM, 1.25 mM, 2.5 mM, 5 mM, and 10 mM. The GC was equipped with the following: a 0.25 μm column (Restek, #RSK-10226), a 10 μL syringe with 0.63mm OD (Shimadzu, Kyoto, Japan; SZ-221-34618-00), and a wool deactivated split glass insert (Shimadzu, Kyoto, Japan; SZ-220-90784-5). The samples were injected at a volume of 1 μL, the column flow rate was set at 1.5 mL/minute at a 20:1 split ratio, helium was the carrier gas. The initial oven temperature of 100 °C/minute was increased to 200 °C, and the detector temperature was set to 300 °C. Samples were run in triplicate, and SCFA concentrations were automatically calculated by comparison to the standard curve.

### 2.10. Statistical Analyses

Unless otherwise specified, all the statistical tests were performed in GraphPad Prism 9 (v9.4.0). All data was subjected to testing for normality (D’Agostino–Pearson omnibus) and homoscedasticity (Brown–Forsythe test (one-way ANOVAs), Spearman’s test (two-way ANOVAs)), and was examined for outliers using the ROUT method (q = 1%). For all data (if multiple factors were present), a two-way ANOVA would be attempted, if the test did not pass normality and/or homoscedasticity, then the data were log transformed, and a new two-way ANOVA was performed. If the data failed to pass both assumptions, a regular one-way ANOVA was attempted, and if the assumptions were not met, the data were log-transformed and was subjected to another one-way ANOVA. If the assumptions were not met, then a non-parametric Kruskal–Wallis test was performed. If there were significant overall effects from the two-way or one-way ANOVAs, then Tukey’s multiple comparison’s test was performed. If the Kruskal–Wallis test was significant, then Dunn’s multiple comparison’s test was performed. All figures show non-transformed data. Detailed statistics for all analyses shown here can be found in Table S9.

## 3. Results

### 3.1. LPS Induces Body Weight Loss and Appetite Suppression, Which Is Not Attenuated by Flaxseed or Flaxseed-Oil Supplemented Diets

Mice BW and DI were assessed during the 3-week period prior to LPS/SAL administration. There were no differences in pre-injection BW between dietary groups (*p* = 0.941) (Figure S1a). Likewise, total diet intake did not differ between groups (*p* = 0.0781) (Figure S1b). 

**Figure 2:**
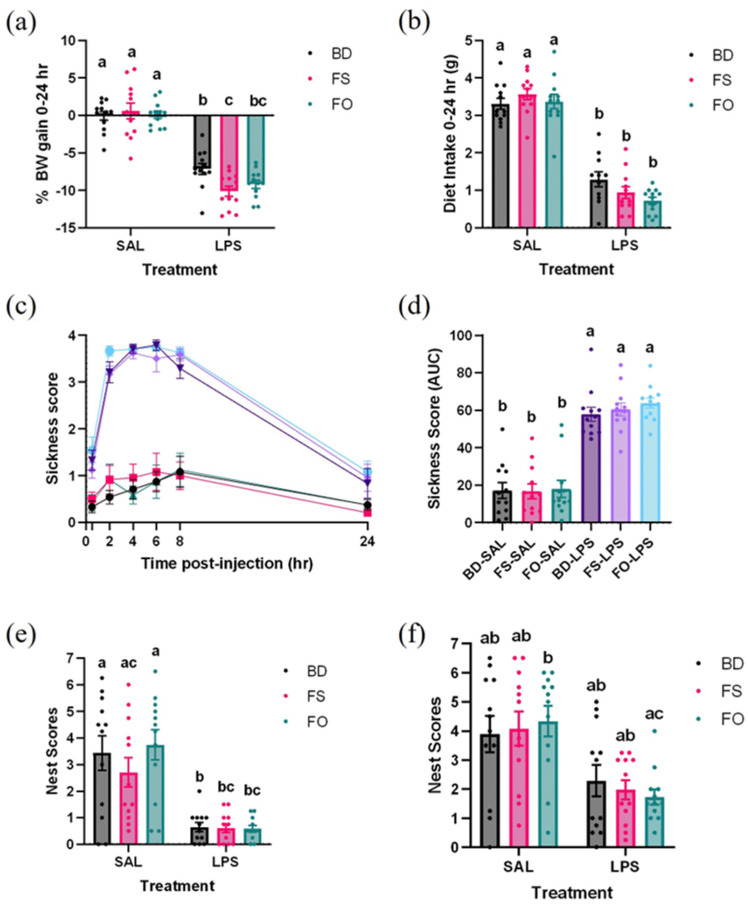
Negative effects of an LPS challenge on body weight, diet intake, and well-being were not improved by FS or FO supplementation. (**a**). Percent body weight loss over 24 h post-injection. (**b**). Diet intake in the 24 h post-injection. (**c**,**d**). Sickness behaviours (**c**). Sickness score over time, (**d**). AUC of sickness scores. (**e**,**f**). Nest quality scores (**e**). Nest scores 8 h post-injection, (**f**). Nest scores 24 h post-injection). Bars not sharing a lowercase letter denotes a significant difference (*p* < 0.05). (*n* = 11–12/group). BD: basal diet. FS: flaxseed; FO: FS oil; LPS: Lipopolysaccharides; SAL: saline.

During the 24 h following injection of SAL or LPS, BW and DI were assessed. There was significantly greater BW loss in the LPS-challenged mice, irrespective of diet, and, notably, the FS-LPS mice lost more weight than the BD-LPS mice (*p* = 0.0436) (Figure 2a). The DI was similarly impacted by LPS, as the LPS-challenged mice ate less food compared to the SAL mice regardless of diet (Figure 2b).

### 3.2. LPS-Induced Decline in Well-Being Was Not Improved by FS or FO Diet Supplementation

Well-being was assessed by measuring sickness behaviours and nest-building activities in mice following SAL or LPS administration. As shown in Figure 2c, sickness behaviours increase during the first 8 h in all LPS-treated mice, and then decline at the 24 h observation point, although not quite reaching the level of the SAL-treated mice. The sickness score AUC showed that LPS treatment, regardless of diet group, increased sickness scores but no other differences between groups were observed (Figure 2d). 

Nest quality was scored at both 8- and 24-h post-injection. At 8 h, all LPS-challenged mice generally had lower quality nests compared to the SAL mice, however there were no differences between the FS-SAL and FS-LPS (*p* = 0.0559) and FO-LPS (*p* = 0.0525) mice (Figure 2e). At 24 h, nest quality scores improved in the LPS-challenged mice, highlighted by the fact there were no differences compared to the SAL-treated mice (*p* > 0.05), with the exception that FO-LPS mice, which continued to have significantly worse nest scores compared to the FO-SAL mice (*p* = 0.0413) (Figure 2f).

### 3.3. Systemic Inflammation Induced by LPS Was Positively Impacted by FS but Not FO Diet Supplementation

**Figure 3:**
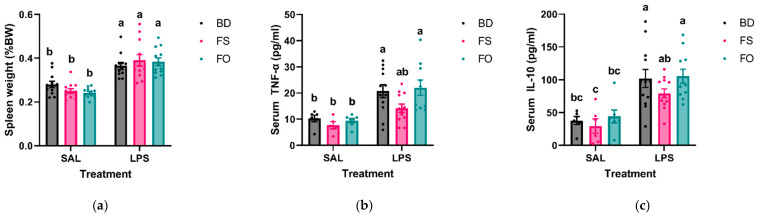
LPS-induced systemic inflammation was partially attenuated in FS- but not FO-fed mice 24 h post-injection. (**a**). Relative spleen weight. (**b**). Serum TNF-α. (**c**). Serum IL-10. Bars not sharing a lowercase letter denotes a significant difference (*p* < 0.05). For serum analytes: SAL (*n* = 5–7/group), LPS (*n* = 10–12/group). Spleen weight (*n* = 12/group). BD: basal diet. FS: flaxseed; FO: FS oil; LPS: Lipopolysaccharides; Sal: saline.

As an overall measure of a systemic inflammatory response to LPS, spleens were collected and weighed. Relative spleen weights highlighted that all LPS-challenged mice had increased spleen weights compared to all SAL-treated mice (*p* < 0.05) (Figure 3a). Considering that many of the negative systemic and central effects of LPS are initiated by circulating pro-inflammatory mediators, we next determined if dietary supplementation with FS or FO could attenuate serum concentrations of select cytokines. Serum concentrations of IL-1β, TNF-α, and IL-10 were measured by multiplex from SAL- and LPS-treated mice. Concentrations of IL-1β were consistently below the minimum detectable limit (4.4 pg/mL), and, thus, were excluded from analysis.

Serum TNF-α and IL-10 were analyzed by one-way ANOVA, which showed differences between groups (*p* < 0.0001). For both analytes, LPS-treated mice fed either BD or FO had elevated serum concentrations compared to all SAL-treated mice (*p* < 0.05). On the other hand, LPS-exposed FS-fed mice had an attenuated response, such that TNF-α concentrations did not differ significantly from all SAL-treated mice, nor BD- or FO-fed mice for IL-10 (*p* > 0.05) (Figure 3b,c). These measures of systemic inflammation highlight an anti-inflammatory effect of FS, but not FO, following a LPS challenge. 

### 3.4. LPS-Induced Neuroinflammation Was Partially Attenuated by FS Supplementation

It is well known that LPS can induce markers of neuroinflammation (IL-1β, TNF-α, IL-10, TLR-4) in the HIP and mPFC of mice [24,25], however, the attenuating role of FS and FO has not yet been investigated. Overall, mice challenged with LPS displayed enhanced neuroinflammation compared to the SAL mice, however, some of the targets were differentially modulated by diet (Table 2). In the HIP, IL-10 and TLR4 mRNA expression were enhanced in all LPS-treated mice compared to their respective SAL controls. On the other hand, LPS-treated mice fed BD or FO had elevated mRNA expression of IL-1β and TNF-α compared to SAL controls, while there was no difference observed in FS-fed mice (Table 2). In the mPFC, IL-1β expression was increased in all the LPS-challenged mice compared to SAL-treated controls, while TLR-4 expression was not altered by LPS. TNF-α expression in the mPFC was significantly increased by LPS in the BD-fed mice, however mice fed FS or FO did not differ from SAL controls. Finally, mPFC IL-10 expression was increased significantly in the LPS-challenged mice fed either BD or FO, however, mice fed FS-supplemented diets did not differ from SAL controls. Taken together, these results show that in the HIP and mPFC, dietary supplementation with FS partially attenuates LPS-induced neuroinflammation, however, minimal effects are observed with FO.

### 3.5. Dietary Supplementation with FS, but Not FO, Alters the Baseline Fecal Microbiota Profile and Activity, Prior to LPS/SAL Administration

Previous studies have shown that consuming FS or isolated FS components can alter the composition and function of the microbiota in humans [35,71] and rodent models [36,40,41,72,73], which may play a role in modulating the response to the LPS challenge. Therefore, we analyzed the fecal microbiota from mice following the three-week dietary intervention, prior to the SAL/LPS challenge, to assess the impact of diet on the baseline microbiota. Fresh fecal samples were collected for 16S rRNA gene sequencing and measurement of SCFA concentrations. 

**Figure 4:**
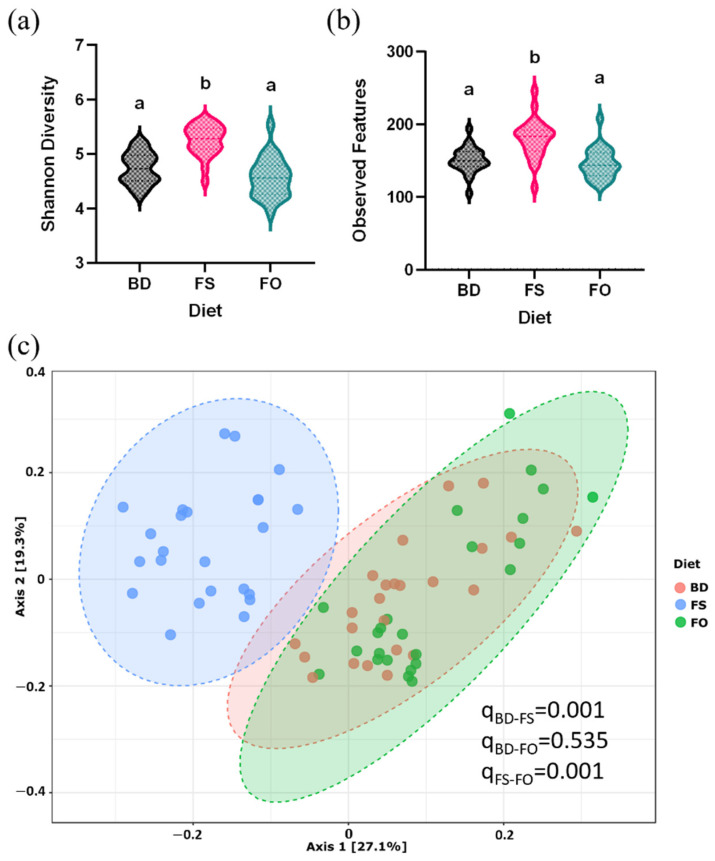
The baseline fecal microbiota diversity is altered in mice consuming FS- but not FO-supplemented diets. (**a**,**b**). Alpha-diversity (**a**). Shannon’s diversity. (**b**). Observed features. (**c**). Beta diversity (Bray–Curtis dissimilarity index). Bars not sharing lowercase letters denotes a significant difference (*p* < 0.05). *n* = 12/group. BD: basal diet. FS: flaxseed; FO: FS oil.

Microbial community α-diversity was assessed by measuring Shannon’s diversity and observed features metrics, and in both, the FS-fed mice had significantly greater α-diversity compared to mice fed BD and FO diets (*p* < 0.0001) (Figure 4a,b). Similarly, β-diversity analyzed by the Bray–Curtis dissimilarity index shows that the mouse samples cluster by diet, such that the diversity of the FS samples is significantly different from both the BD and FO mice (q = 0.0015); however, there are no differences between BD and FO in this metric (Figure 4c).

**Figure 5:**
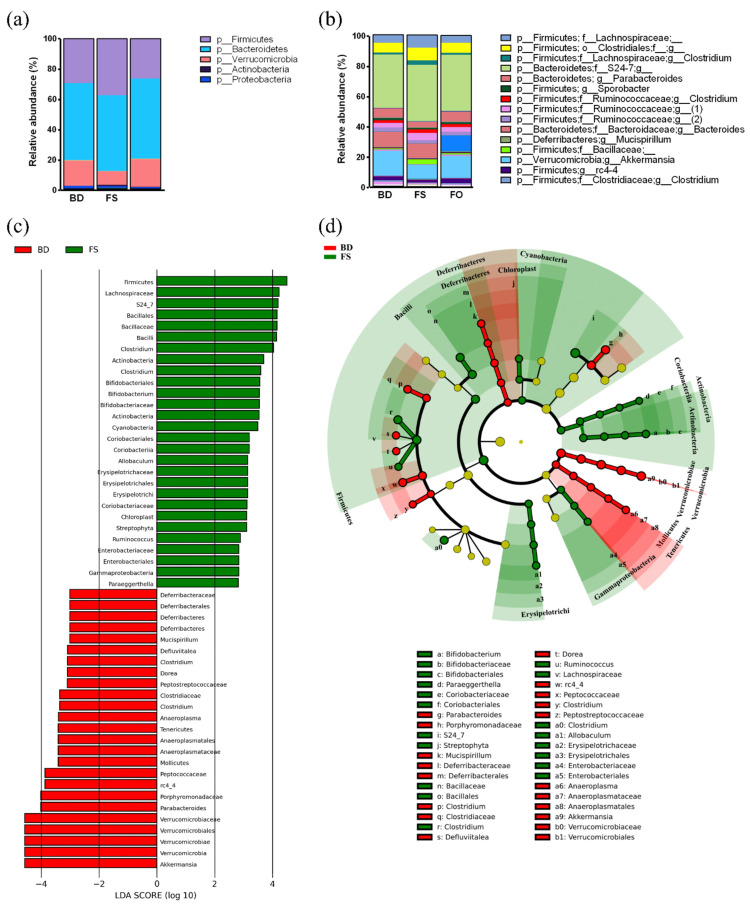
The baseline composition of the fecal microbiota is altered in FS- but not FO-fed mice. (**a**,**b**). Relative abundance of the fecal microbiota at the (**a**). phylum, and (**b**). genus level. (**c**). LEfSE bar plot denoting the significantly different clades between the BD- and FS-fed mice. (**d**). Cladogram depicting the significantly different clades from (**c**). The colours denote a significantly increased clade in the BD-fed mice (red), and in the FS-fed mice (green), yellow denotes no significant changes. BD: basal diet. FS: flaxseed; FO: FS oil.

The fecal microbiota composition, prior to LPS/SAL administrations, at both the phylum and genus level are shown in Figure 5a,b; Table S5. As expected, the microbial community was primarily comprised of members from the Bacteroidetes, Firmicutes, and Verrucomicrobia phyla. To further visualize the fecal microbiota, a cladogram representation of the structure and a bar graph of the dominant bacteria were made and shown in Figure 5c,d. Compared to BD-fed mice, 3 week FO dietary supplementation did not alter the microbial composition at any taxonomic level (Table S5). On the other hand, FS-fed mice had greater relative abundance of 3 phyla compared to BD-fed mice (Firmicutes, Actinobacteria, Cyanobacteria) and reduced relative abundance of Verrucomicrobia, Tenericutes, and Deferribacteres. In total, there were 28 clades that were greater in the FS-fed mice, and 25 that were greater in the BD-fed mice (Figure 5c). FS dietary supplementation resulted in increases in S24-7, Lachnospiraceae, *Bifidobacterium*, Coriobacteriaceae, Enterobacteriaceae, and Streptophyta, to name a few. On the contrary, compared to the BD group, FS-fed mice had reduced relative abundance of *rc4-4*, *Dorea*, *Defluviitalea*, *Parabacteroides*, *Anaeroplasma*, *Mucispirillum*, and *Akkermansia*. Therefore, 3 week dietary supplementation with FS, but not FO, altered the fecal microbiota diversity and composition, prior to LPS or SAL administration, compared to the BD-fed mice.

**Figure 6:**
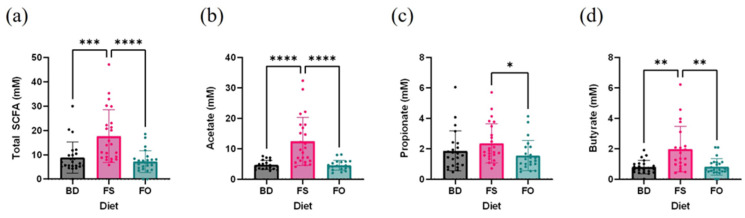
FS but not FO dietary supplementation increased fecal SCFA concentration. (**a**–**d**). Fecal SCFA concentrations (**a**). Total SCFA, (**b**). acetate, (**c**). propionate, (**d**). butyrate. * = *p* < 0.05, ** = *p* < 0.01, *** = *p* < 0.001, **** = *p* < 0.0001 (*n* = 20–24/diet). Detailed statistics can be found in Table S9. BD: basal diet. FS: flaxseed; FO: FS oil.

Diet-induced changes in microbiota community function were assessed by measuring the concentrations of short-chain fatty acids (SCFAs) that were extracted from feces collected prior to SAL and LPS injection, and quantified using gas chromatography. There were differences in the total SCFAs between diets, such that FS-fed mice had higher concentrations compared to the BD-fed (*p* = 0.004) and FO-fed mice (*p* < 0.0001) (Figure 6a). Specifically, FS-fed mice had higher concentrations of acetate (Figure 6b) and butyrate (Figure 6d). Fecal propionate was increased in the FS-fed mice compared to FO- (*p* = 0.0290) but not BD-fed mice (*p* = 0.1748) (Figure 6c). Collectively, these results demonstrate that prior to LPS exposure, three weeks of dietary supplementation with FS significantly alters the baseline microbiota composition and function compared to both BD- and FO-fed mice. 

### 3.6. LPS-Challenged Mice Display Altered Fecal Microbiota Diversity and Composition in a Diet Dependent Manner 

LPS exposure (i.p) has been shown to induce microbial dysbiosis in several animal models, including C57Bl/6 mice [26,27,28,29]. Since this shift in microbial community could play a role in the peripheral and central effects of LPS, we next set out to determine if LPS-induced dysbiosis differed in mice fed different background diets (BD, FS, FO). Further, since mice pre-fed FS- but not FO-supplemented diets altered the microbial community structure and function prior to LPS challenge (Figure 4, Figure 5 and Figure 6), we focused our analyses to compare LPS- and SAL- exposed mice within each dietary group (e.g., FS-SAL vs. FS-LPS), as opposed to between diet group comparisons (e.g., BD-SAL vs. FS-LPS). 

**Figure 7:**
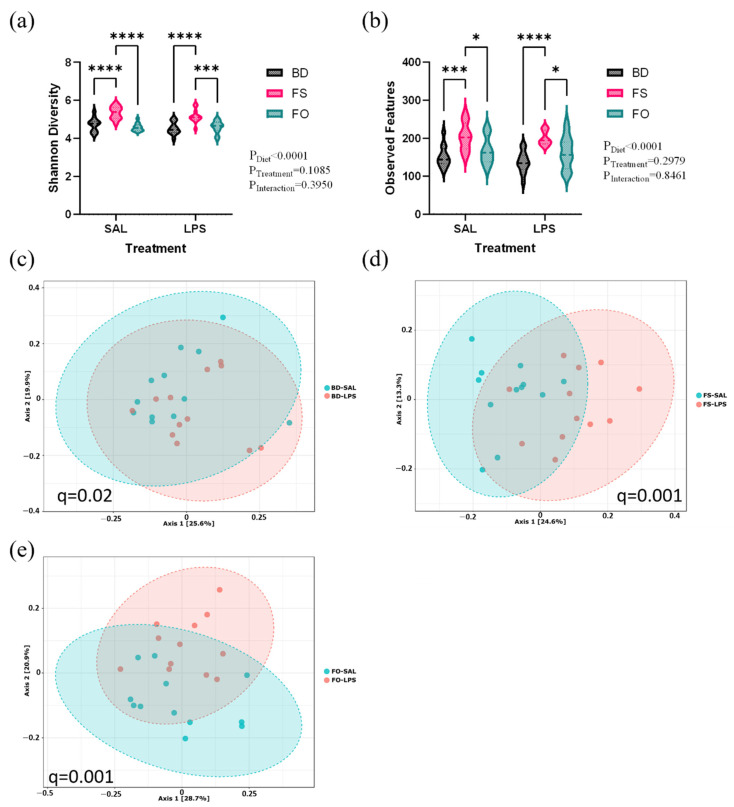
The fecal microbiota diversity is altered in LPS-challenged C57Bl/6 mice. (**a**,**b**). Alpha-diversity (**a**). Shannon diversity, (**b**). observed features. (**c**–**e**). Bray–Curtis β-diversity visualized by PCoA (**c**). BD SAL vs. LPS, (**d**). FS SAL vs. LPS, (**e**). FO SAL vs. LPS. * = *p* < 0.05, *** = *p* < 0.001, **** = *p* < 0.0001 (*n* = 12/group). BD: basal diet. FS: flaxseed; FO: FS oil; LPS: Lipopolysaccharides; Sal: saline.

Alpha diversity (Shannon’s and observed features) was assessed by two-way ANOVA, which demonstrated an overall effect of diet (*p* < 0.001), such that FS-fed mice had increased diversity, however, there was no effect of treatment (i.e., LPS). These results indicate that 24 h post-LPS injection, there were no LPS-induced changes in microbial community diversity, however, the FS-induced increase in α-diversity observed prior to LPS/SAL exposure (Figure 7a,b) persisted. On the other hand, analyses of fecal microbial community β-diversity highlighted LPS-induced effects, which may be background diet-dependent. As shown in Figure 7c–e, the Bray–Curtis dissimilarity index shows that LPS induces a shift in β-diversity in all diet groups, however, the effect is more prominent in the FS- (FS-SAL vs. FS-LPS) (Figure 7d) and FO-fed mice (FO-SAL vs. FO-LPS) (Figure 7e) compared to the BD-fed mice (BD-SAL vs. BD-LPS) (Figure 7c).

**Figure 8:**
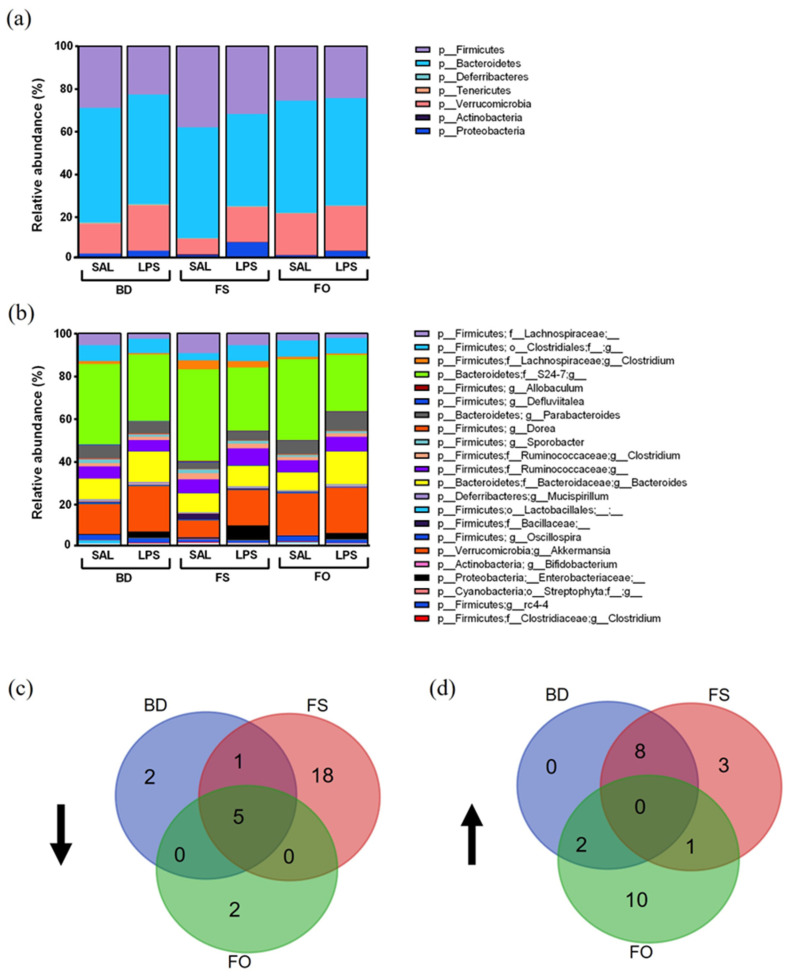
The fecal microbiota composition is impacted by LPS-challenge in a diet-dependent manner. (**a**,**b**). Bar plots showing the (**a**) phylum and (**b**) genus level relative abundances in each dietary group 24 h post SAL or LPS injection. (**c**,**d**). Venn diagrams depicting the taxonomic changes induced by LPS across the three dietary groups. (**c**) Decreased in the LPS groups. (**d**). Increased in the LPS groups. The specific taxa associated with the Venn diagram, and the corresponding LEfSE plots, can be found in Figure S3 (*n* = 12/group). BD: basal diet. FS: flaxseed; FO: FS oil; LPS: Lipopolysaccharides; Sal: saline.

To gain a better understanding of the community members driving the LPS-induced change in β-diversity, the relative abundance of members of the microbial community at both the phylum and genus level were examined (Figure 8a,b). The microbiota composition was visualized by a cladogram representation with dominant bacteria highlighted (Figure S3). In Figure 8d,e, we show a Venn diagram depicting the shared/unique effects of LPS on mice fed different diets. Notably, in LPS-challenged mice, there were 5 shared clades reduced across diets including S24-7, Lachnospiraceae, Lactobacillales, Clostridium, and Clostridia, compared to SAL-treated controls (Figure 8d and Figure S3, Table S7). FS-fed mice had the greatest number (18) of LPS-induced decreases in relative abundance in the fecal microbiota including Coriobacteriaceae, Bacteroidetes, Streptophyta, and Actinobacteria. 

The FO-fed and the BD-fed mice only had two unique changes with decreases in *rc4-4* and *Peptococcaceae* in the FO-fed mice, and decreases in *Defluviitalea* and Ruminococcaceae in the BD-fed mice. There were no commonly increased clades following an LPS challenge, however, BD- and FS-fed mice shared eight similarities including increased Enterobacteriaceae and *Akkermansia* (Figure 8e, Table S8). The FO-fed mice had the greatest number of clades (10) increased by LPS including Deferribacteres, *Parabacteroides*, and *Muscispirillum*, whereas FS-fed mice only had three unique clades (Proteobacteria, *Ruminococcus*, and Ruminococcaceae) increased by LPS. These results highlight that the effects of LPS on the fecal microbiota are dependent on the background diet, however, there are certain taxa that are consistently altered, which may be targets of future research.

**Figure 9:**
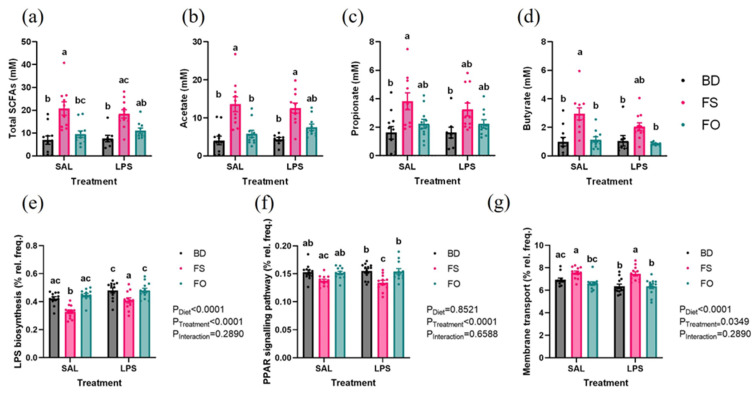
Measured and inferred function of the fecal microbiota post LPS challenge. (**a**–**d**). Fecal SCFAs. (**a**). Total SCFAs, (**b**). acetate, (**c**). propionate, (**d**). butyrate (*n* = 10–12/group). (**e**–**g**). Inferred function of the fecal microbiota (**e**). LPS biosynthesis, (**f**). PPAR signalling pathway, (**g**). membrane transport (*n* = 23–24/group). Bars that share letters are not significantly different. BD: basal diet. FS: flaxseed; FO: FS oil; LPS: Lipopolysaccharides; Sal: saline.

The measured fecal microbiota function was assessed by measuring concentrations of SCFAs from feces collected 24 h post injection of SAL or LPS. The total SCFA concentration in the FS-fed mice was increased compared to BD-fed mice regardless of treatment (Figure 9a–d). FS-SAL had higher total SCFA than FO-SAL, but this difference was not seen between FS-LPS and FO-LPS. Similarly, acetate was significantly increased in FS-fed mice, regardless of diet, however, FO-LPS did not differ from FS-LPS. Propionate only differed between FS-SAL and BD-fed mice regardless of treatment. Butyrate concentrations were significantly increased in the FS-fed mice compared to the BD- and FO-fed mice, but only in the mice who received an injection of SAL.

The function of the fecal microbiota was inferred using the PICRUST2 pipeline in QIIME2. Similar to the effects of LPS on the fecal microbiota composition, the inferred function is reliant on the background diet. In BD-fed mice, there were no significant pathway differences between SAL- and LPS-challenged mice. On the contrary, in the FS-fed group, there were 132 pathway differences and 3 differences in the FO-fed mice (Figure S4). Multiple group analysis reveals significant effects of an LPS-challenge, including increased LPS biosynthesis, PPAR signalling pathway, and decreased membrane transport as a few examples (Figure 9e–g). Notably, there were diet-specific effects on LPS biosynthesis and membrane transport, with decreased relative frequency in the FS-fed mice regardless of treatment. (Figure 9e,g). Taken together, both the measured and inferred functions of the fecal microbiota are altered in the FS-fed mice, which could be critical in the context of the mGBA, and especially the partial attenuation of LPS-induced inflammation.

## 4. Discussion

The objectives of this study were to determine the effects of dietary supplementation with FS and FO on the LPS-induced changes across the mGBA. Overall, this study shows that LPS induces negative effects throughout the mGBA, specifically through changes in the composition of the gut microbiota and increases in systemic and neuro-inflammation. These changes were differentially impacted by dietary supplementation, such that mice fed a flaxseed-supplemented diet displayed a partial attenuation of the proinflammatory effects of LPS, together with robust changes to the fecal microbial community structure and function. On the other hand, in mice consuming FO, limited effects were observed across the mGBA. This may suggest that the beneficial effects of FS seen in this study were not solely driven by its oil content, but may be mediated by other bioactives (or their combinations) present in the whole seed (e.g., fibre, lignans). 

An acute LPS exposure in mice fed BD, induced inflammation both systemically and centrally, which aligned with previous studies [21,24,25,38,74,75]. Specifically, following LPS exposure, mice experienced sickness behaviours (Figure 2c,d), altered nest-building activity (Figure 2e,f), elevated serum IL-10 and TNF-α (Figure 3b,c), and enhanced inflammatory gene expressions (IL1β, TLR-4, IL-10, and TNF-α) in the HIP and/or mPFC (Table 2). In our samples, IL-1β is not detected in the serum of LPS- or SAL-treated mice 24 h post-injection, which may be explained by findings showing that IL-1β is an early inflammatory response (peak elevation at 4–6 h post-LPS exposure), which returns to control levels by 24 h post-injection [76]. 

Furthermore, there was evidence of microbial dysbiosis following LPS exposure, as evidenced by a change in microbial β-diversity and altered relative abundances of specific microbial groups (e.g., increased Enterobacteriaceae and Bacteroides, and reduced S24-7, Lachnospiraceae, Lactobacillales, and Clostridium). These findings agree, in part, with previous studies showing that LPS exposure (i.p) can induce microbial dysbiosis resulting in increased abundance of potentially pathogenic members throughout the intestine, such as Enterobacteriaceae [26,28,77], which may have contributed to enhanced inflammation. On the other hand, despite microbial compositional changes, in BD-fed mice treated with LPS, there was no evidence of reduced alpha diversity or altered microbial activity as measured by SCFA concentrations or inferred function of the metagenome (Figure 7 and Figure 9). This may be related to the dose of LPS used, the baseline microbiota composition, and the BD nutritional composition, as well as the timing and anatomical location of sample collection post-LPS injection. In a previous study, 4–6-week-old male C57Bl/6 mice were subjected to a 10 mg/kg dose of LPS (i.p) and 24 h post-injection, the composition and activity of the cecal microbiota was assessed. Consistent with our results, an increased relative abundance of Enterobacteriaceae following an LPS challenge was noted; however, they also observed a reduction in SCFA concentrations [28]. The dose of LPS (10 mg/kg/BW vs. 1 mg/Kg BW), the sample source (cecal vs. fecal), as well as the composition of the diet used (standard chow vs. purified AIN-93G), are potential sources of differentiation between studies, so comparisons should be made with some degree of caution. In another study, the fecal microbiota composition of 8-week-old male C57Bl/6 mice was examined ~24-h after a 0.5 mg/kg LPS (i.p), results of which showed an LPS-induced reduction in α-diversity, in addition to modulation to the structure of the microbial community (e.g., enhanced Enterobacteriales and Bacteroidetes) [26]. Despite variations in some of the outcomes observed between studies, our results support the findings that LPS exposure can induce negative impacts on the intestinal microbiota, which may contribute to its proinflammatory effects systemically and centrally.

Importantly, our results also suggest that dietary supplementation with FS, and less so FO, can attenuate some of the negative effects induced by LPS across the mGBA. Specifically, consumption of FS, but not FO, for 3 weeks prior to and during an acute LPS challenge, can partially attenuate the LPS-induced increase in serum concentrations of TNF-α and IL-10 (Figure 3b,c). Similarly, in both the HIP and/or mPFC, the LPS-induced increase in relative gene expression of cytokines (TNF-α, IL-10, and IL-1β) was attenuated in mice fed FS, with minimal attenuating effects observed in mice fed FO (Table 2). The fecal microbiota was also examined prior to SAL/LPS injection to determine if diet supplementation altered the baseline microbial community structure, which may play a role in attenuating the LPS inflammatory response. Despite a general lack of effect of FO, our results confirm previous findings [36,41] that supplementation of diet with ground flaxseed can induce robust changes to the microbiota community structure (enhanced microbial diversity and altered microbial relative abundances) and function (e.g., enhanced production of SCFA concentrations), which may play a role in attenuating inflammation-driven disease processes. For example, as also shown by Taibi et al., our findings indicate that FS can enhance the abundance of Actinobacteria and its family Coriobacteriaceae (Tables S5 and S6). Coriobacteriaceae can metabolize the lignan SDG, which is enriched in flaxseed, resulting in production of microbial-derived metabolites, ED and EL, which have known anti-inflammatory and antioxidant properties [43,44]. Importantly, SDG has been shown to attenuate LPS-induced BBB permeability and reduce neuroinflammation in mice [45], and, thus, serve as an important flaxseed bioactive, which may explain some of the anti-inflammatory effects observed in our current study.

Interestingly, we also observed a significant reduction in the relative abundance of *Akkermansia muciniphila*, a mucin-associated and acetate and propionate producer [78,79] following 3-week dietary supplementation with FS, a result that has been previously found in the fecal and/or cecal microbiota of male C57Bl/6 mice fed FS-based diets [36,39]. This result remains intriguing, since a decrease in *A. muciniphila* is generally associated with increased risk of metabolic-related diseases, such as diabetes and obesity [80], while increased abundance has been observed in inflammatory diseases, such as multiple sclerosis, where it is has been associated with a proinflammatory environment [81,82]. Interestingly, this inhibitory effect of FS on *A. muciniphila* was negated following LPS exposure (Figure 8, Table S6). On the other hand, specific FS-increased taxa, such as Lachnospiraceae and *Bifidobacterium*, may suggest a beneficial role of FS. Lachnospiraceae are known to produce SCFAs, which can counteract LPS-induced inflammation. Similarly, *Bifidobacterium* (some of which are used as probiotics), may induce beneficial effects centrally, as they have been shown to ameliorate phenotypes indicative of mental illnesses in rodents [83,84]. Some of these beneficial taxonomic changes may translate to anti-inflammatory effects systemically and in the brain, such as reduced inflammatory signaling by glial cells and increased BBB integrity [85].

In addition, FS-fed mice had enhanced microbial diversity and activity (Figure 4 and Figure 6), which may have contributed to its anti-inflammatory effects following LPS exposure. Flaxseed is rich in fermentable non-digestible fibers, which generate an increase in microbial-derived SCFAs, notably acetate and butyrate (Figure 6 and Figure 9a–d). Previous studies have demonstrated anti-inflammatory effects of SCFAs, both systemically and centrally, which may be related to disruption of the LPS-TLR-4 signalling cascades [86,87]. Further, previous studies utilizing butyrate supplementation show a reduction in LPS-induced inflammation in the HIP (Balb/c mice) [88] and in the PFC [23]. Thus, the potential benefits of a single SCFA may suggest the importance of the increased SCFAs, especially butyrate, in the FS-fed mice. Overall, these data suggest that FS, but not FO, attenuates LPS-induced systemic and neuroinflammation, however, the bioactives responsible for this effect (i.e., lignans, fermentable fiber) requires further exploration. Importantly, these beneficial FS-induced changes (increased relative abundance of Lachnospiraceae and *Bifidobacterium,* and elevated concentration of SCFAs) remain elevated in FS-fed mice following LPS exposure (Figure 8, Table S6), which may suggest that the priming effect of FS on the microbiota prior to an LPS-challenge may contribute to its anti-inflammatory effects.

In this study, we examined the effects of dietary supplementation with FS and FO on the LPS-induced changes across the GBA. Overall, FS, but not FO, partially attenuated some negative effects of LPS, including reduced systemic and neuroinflammation, which may be related to beneficial changes in the structure and function of the microbiota. Although further studies are required, these findings highlight the potential for a dietary component to attenuate inflammatory processes related to increased mental health disorders. The limited impact of FO on outcomes measured in this study differs from previous findings [42], which may be related to sex, rodent model used, or the cultivar of FS from which FO was extracted (e.g., different fatty acid profiles). Since dietary supplementation with FO had very limited effect throughout this study, this suggests that the beneficial effects of FS are either independent of the oil content, or the effects are due to a combination of different bioactives.

## Figures and Tables

**Table 1:** Experimental diet composition.

Ingredients	BD (g/Kg)	BD + 10% FS (g/Kg)	BD + 4% FO (g/Kg)
Casein	200	173	200
L-Cystine	3	3	3
Corn starch	397.49	387.22	397.48
Maltodextrin	132	132	132
Sucrose	100	100	100
Corn oil	70	30.5	30.3
Cellulose	50	19.4	50
Mineral mix (AIN-93G-MX)	35	35	35
Vitamin mix (AIN-93-VX)	10	10	10
Choline bitartrate	2.5	2.5	2.5
TBHQ ^a^	0.014	0.014	0.014
Ground flaxseed	0	100	0
Flaxseed oil	0	0	39.7
Caloric density (Kcal/g)	3.8	3.7	3.8

BD: basal diet. FS: flaxseed; FO: FS oil. ^a^ Tertiarybutylhydroquinon.

**Table 2:** The effects of FS and FO on LPS-induced mRNA expression of inflammation biomarkers in the hippocampus and medial prefrontal cortex.

Site	Target	SAL			LPS		
		BD	FS	FO	BD	FS	FO
HIP	IL-1β	0.31 ± 0.053 ^c^	0.49 ± 0.067 ^bc^	0.33 ± 0.041 ^c^	10.59 ± 1.8 ^a^	9.13 ± 1.1 ^ab^	17.79 ± 2.6 ^a^
	TNF-α	14.14 ± 3.69 ^c^	28.79 ± 6.43 ^bc^	23.65 ± 3.53 ^bc^	69.78 ± 15.86 ^a^	60.85 ± 14.13 ^ab^	75.35 ± 10.43 ^a^
	IL-10	0.71 ± 0.15 ^b^	1.11 ± 0.26 ^b^	0.97 ± 0.18 ^b^	4.63 ± 0.67 ^a^	3.41 ± 0.59 ^a^	3.16 ± 0.47 ^a^
	TLR-4	9.19 ± 0.55 ^bc^	8.67 ± 0.8 ^c^	7.73 ± 0.57 ^c^	13.15 ± 1.29 ^a^	12.77 ± 0.88 ^a^	11.92 ± 0.8 ^ab^
mPFC	IL-1β	0.94 ± 0.11 ^b^	0.9 ± 0.13 ^b^	0.84 ± 0.14 ^b^	22.15 ± 3.61 ^a^	14.53 ± 3.47 ^a^	17.78 ± 2.38 ^a^
	TNF-α	15.95 ± 5.11 ^b^	16.72 ± 5.8 ^b^	13.71 ± 5.12 ^b^	56.42 ± 10.53 ^a^	40.22 ± 8.75 ^ab^	25.3 ± 5.43 ^ab^
	IL-10	1.16 ± 0.17 ^b^	1.22 ± 0.3 ^b^	1.41 ± 0.3 ^b^	2.9 ± 0.36 ^a^	1.96 ± 0.3 ^ab^	2.92 ± 0.4 ^a^
	TLR-4	17.65 ± 1.98	14.54 ± 1.69	20.12 ± 2.3	20.03 ± 2.04	16.43 ± 1.61	17.21 ± 1.25

Relative mRNA expression (mean ± SEM) of inflammatory targets in the HIP and mPFC 24 h post-injection of SAL or LPS. Values that do not share a lowercase letter are significantly different. *n* = 9–12/group. Detailed statistics can be found in Table S9. BD: basal diet. FS: flaxseed; FO: FS oil; LPS: Lipopolysaccharides; Sal: saline.

## Data Availability

Additional data from this study will be available upon request to the corresponding author.

## Supplementary Materials

The following supporting information can be downloaded at: https://www.mdpi.com/article/10.3390/nu15163542/s1, Table S1: Macronutrient composition of whole ground flaxseed. Table S2: Fatty acid profile of extracted flaxseed oil. Table S3: Scoring criteria for nest quality. Table S4: Primer sequences. Table S5: The relative abundance of the fecal microbiota pre-injection. Table S6: The relative abundance of the fecal microbiota after SAL/LPS injection. Table S7: Taxonomic clades increased by an LPS challenge. Table S8: Taxonomic clades decreased by an LPS challenge. Table S9: Detailed statistics. Figure S1: BW and total DI pre-injection. Figure S2: Representative images of nest quality scores. Figure S3: Lefse bar plots and cladograms for comparing SAL/LPS within diet. Figure S4: Inferred functional changes with LPS.
