# Supplementary material for: Dietary Flaxseed and Flaxseed Oil Differentially Modulate Aspects of the Microbiota Gut–Brain Axis Following an Acute Lipopolysaccharide Challenge in Male C57Bl/6 Mice
Source: Nutrients. 2023 Aug 11;15(16):3542. doi: 10.3390/nu15163542 (PMC10459276; doi:10.3390/nu15163542)
Supplement: Supplementary file 1 [file nutrients-15-03542-s001.zip › Supplemental Tables and Figures.pdf]

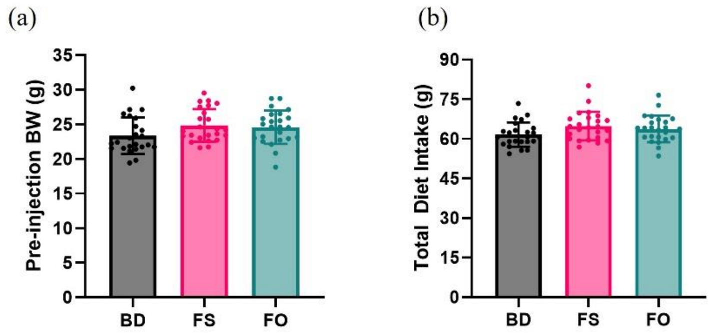

Figure S1: BW (A) and total diet intake (B) in mice following 3 weeks of diet consumption.

(BD= basal diet; FS = 10% FS diet; FO = 4% FO diet) (n=24/group).

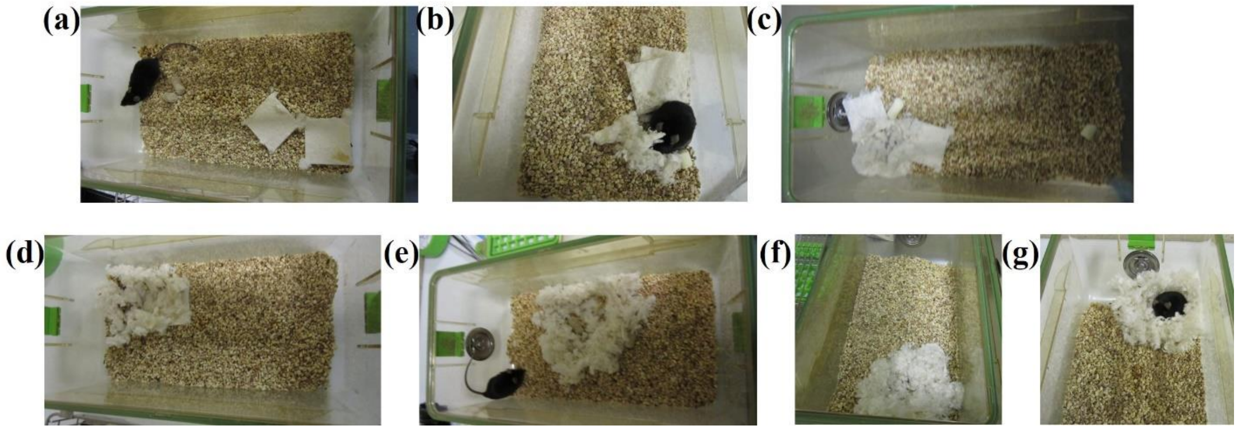

Figure S2: Representative pictures of nest quality scores.

A-G. Nest scores (0, 1, 2, 3, 4, 5, 6, 6.5). Scoring system can be found in Table S3.

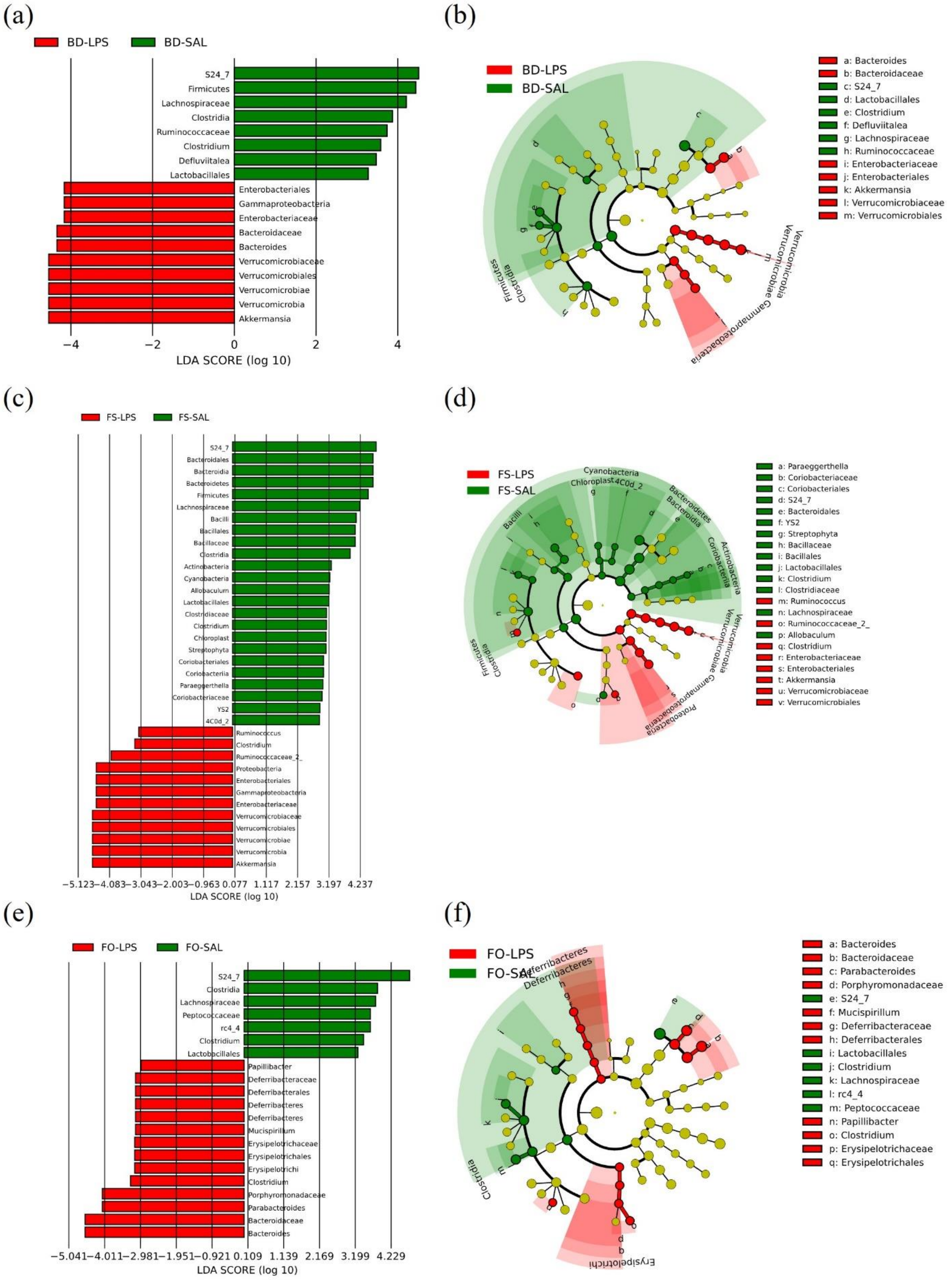
(a)
BD-LPS
BD-SAL
S24_7
Firmicutes
Lachnospiraceae
Clostridia
Ruminococcaceae
Clostridium
Defluviitalea
Lactobacillales
Enterobacteriales
Gammaproteobacteria
Enterobacteriaceae
Bacteroidaceae
Bacteroides
Verrucomicrobiaceae
Verrucomicrobiales
Verrucomicrobiae
Verrucomicrobia
Akkermansia
−4
−2
0
2
4
LDA SCORE (log 10)
(b)
BD-LPS
BD-SAL
a: Bacteroides
b: Bacteroidaceae
c: S24_7
d: Lactobacillales
e: Clostridium
f: Defluviitalea
g: Lachnospiraceae
h: Ruminococcaceae
i: Enterobacteriaceae
j: Enterobacteriales
k: Akkermansia
l: Verrucomicrobiaceae
m: Verrucomicrobiales
Firmicutes
Clostridia
Verrucomicrobia
Verrucomicrobiae
Gammaproteobacteria
(c)
FS-LPS
FS-SAL
S24_7
Bacteroidales
Bacteroidia
Bacteroidetes
Firmicutes
Lachnospiraceae
Bacilli
Bacillales
Bacillaceae
Clostridia
Actinobacteria
Cyanobacteria
Allobaculum
Lactobacillales
Clostridiaceae
Clostridium
Chloroplast
Streptophyta
Coriobacteriales
Coriobacteriia
Paraeggerthella
Coriobacteriaceae
YS2
4C0d_2
Ruminococcus
Clostridium
Ruminococcaceae_2_
Proteobacteria
Enterobacteriales
Gammaproteobacteria
Enterobacteriaceae
Verrucomicrobiaceae
Verrucomicrobiales
Verrucomicrobiae
Verrucomicrobia
Akkermansia
−5.123
−4.083
−3.043
−2.003
−0.963
0.077
1.117
2.157
3.197
4.237
LDA SCORE (log 10)
(d)
FS-LPS
FS-SAL
a: Paraeggerthella
b: Coriobacteriaceae
c: Coriobacteriales
d: S24_7
e: Bacteroidales
f: YS2
g: Streptophyta
h: Bacillaceae
i: Bacillales
j: Lactobacillales
k: Clostridium
l: Clostridiaceae
m: Ruminococcus
n: Lachnospiraceae
o: Ruminococcaceae_2_
p: Allobaculum
q: Clostridium
r: Enterobacteriaceae
s: Enterobacteriales
t: Akkermansia
u: Verrucomicrobiaceae
v: Verrucomicrobiales
Cyanobacteria
Chloroplast
4C0d_2
Bacteroidetes
Bacteroidia
Actinobacteria
Coriobacteriia
Bacilli
Firmicutes
Clostridia
Verrucomicrobia
Verrucomicrobiae
Gammaproteobacteria
Proteobacteria
(e)
FO-LPS
FO-SAL
S24_7
Clostridia
Lachnospiraceae
Peptococcaceae
rc4_4
Clostridium
Lactobacillales
Papillibacter
Deferribacteraceae
Deferribacterales
Deferribacteres
Deferribacteres
Mucispirillum
Erysipelotrichaceae
Erysipelotrichales
Erysipelotrichi
Clostridium
Porphyromonadaceae
Parabacteroides
Bacteroidaceae
Bacteroides
−5.041
−4.011
−2.981
−1.951
−0.921
0.109
1.139
2.169
3.199
4.229
LDA SCORE (log 10)
(f)
FO-LPS
FO-SAL
a: Bacteroides
b: Bacteroidaceae
c: Parabacteroides
d: Porphyromonadaceae
e: S24_7
f: Mucispirillum
g: Deferribacteraceae
h: Deferribacterales
i: Lactobacillales
j: Clostridium
k: Lachnospiraceae
l: rc4_4
m: Peptococcaceae
n: Papillibacter
o: Clostridium
p: Erysipelotrichaceae
q: Erysipelotrichales
Deferribacteres
Deferribacteres
Clostridia
Erysipelotrichi

Figure S3. Lefse bar plots and cladograms for comparing SAL/LPS within diet.

A. LEfSE bar plot denoting the significantly different clades between the BD-SAL and BD-LPS mice. B. Cladogram depicting the significantly different clades from A. C. LEfSE bar plot denoting the significantly different clades between the FS-SAL and FS-LPS mice. D. Cladogram depicting the significantly different clades from C. E. LEfSE bar plot denoting the significantly different clades between the FO-SAL and FO-LPS mice. F. Cladogram depicting the significantly different clades from E. The cladogram colours denote a significantly increased clade in the LPS-challenged mice (red), and in the SAL-challenged mice (green), yellow denotes no significant changes. The diameter of the circle denotes the size of the taxon.

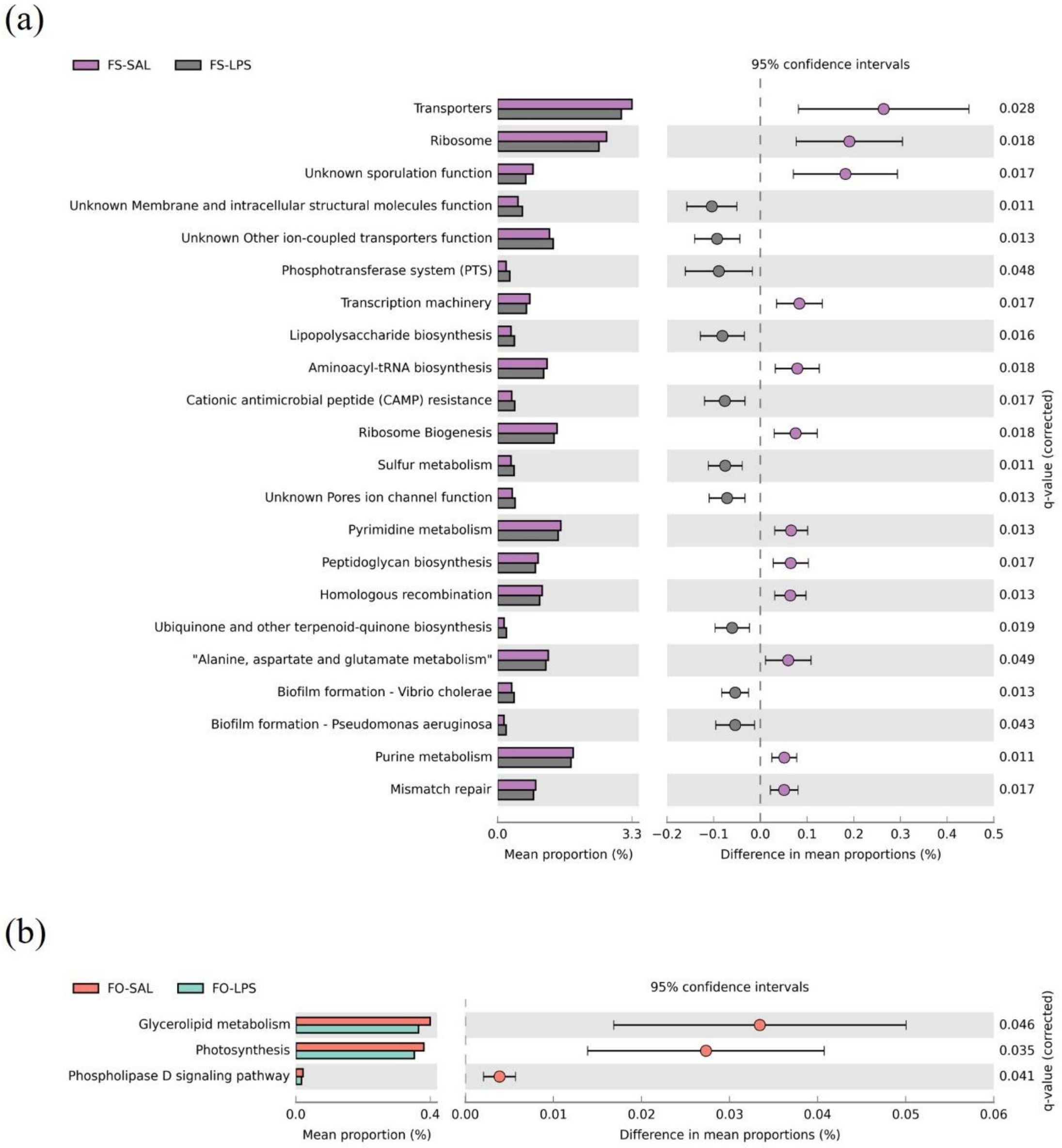

Figure S4. The inferred functional changes with LPS are diet dependent.

A. FS-fed mice had the greatest number of functional changes following a LPS-challenge. B. FO-fed mice had fewer inferred functional changes in the microbiota post-injection of LPS. An effect size filter was applied to the FS- fed mice to narrow the functions down from 132.

Supplemental Tables

Table S1: Macronutrient composition of whole ground flaxseed

Nutritional parameter	Content (g/100g)	Method
Protein	17.08	AOAC 992.15
Available carbohydrates	3.6	Calculation
Fat	39.7	AOAC 996.06
Saturated fatty acids	3.89	AOAC 996.06
Cis-MUFAs	6.87	AOAC 996.06
Cis-PUFAs	27.1	AOAC 996.06
Trans-fatty acids	0.13	AOAC 996.06
Omega-3 PUFAs	20.7	AOAC 996.06
Omega-6 PUFAs	6.40	AOAC 996.06
Total dietary fibre	30.6	AOAC 991.43, 985.29
Soluble dietary fibre	9.7	AOAC 991.43
Insoluble dietary fibre	20.9	Calculation
Moisture	5.53	AOAC\ADPI methods
Ash	3.5	AOAC 923.03
Energy (kcal)	501	Calculation

Table S2: Fatty acid profile of extracted flaxseed oil

Common Name	Systematic Name	Lipid numbers	Fatty acid content	
			**%**	**mg/g**
Butyric acid	Butanoic acid	**C4:0**	0.00	0.00
Caproic acid	Hexanoic acid	**C6:0**	0.00	0.00
Caprylic acid	Octanoic acid	**C8:0**	0.47	4.52
Capric acid	Decanoic acid	**C10:0**	0.01	0.06
Lauric acid	Dodecanoic acid	**C12:0**	0.01	0.14
Myristic acid	Tetradecanoic acid	**C14:0**	0.19	1.78
Myristoleic acid	Tetradecenoic acid	**C14:1**	0.00	0.00
Pentadecylic acid	Pentadecanoic acid	**C15:0**	0.01	0.13
Palmitic acid	Hexadecanoic acid	**C16:0**	5.46	52.63
Palmitoleic acid	Hexadecenoic acid	**C16:1**	0.07	0.63
Stearic acid	Octadecanoic acid	**C18:0**	3.98	38.30
Oleic acid	Octadecenoic acid	**C18:1 (C9)**	16.64	160.30
Vaccenic acid	Octadecenoic acid	**C18:1 (C11)**	0.83	7.99
Linoleic acid	Octadecadienoic	**C18:2n6**	15.97	153.82
γ-Linolenic acid	Octadecatrienoic acid	**C18:3n6**	0.00	0.00
α-linolenic acid (ALA)	Octadecatrienoic acid	**C18:3n3**	55.03	530.17
Stearidonic acid	Octadecatetraenoic acid	**C18:4n3**	0.00	0.00
Arachidic acid	Icosanoic acid	**C20:0**	0.16	1.51
Gadoleic acid	Eicosenoic acid	**C20:1 (C7)**	0.04	0.39
Gondoic acid	Eicosenoic acid	**C20:1 (C9)**	0.14	1.36
Paullinic acid	Eicosenoic acid	**C20:1 (C11)**	0.02	0.19
Eicosadienoic acid	Eicosadienoic acid	**C20:2n6**	0.08	0.75
Dihomo-γ-linolenic acid (DGLA)	Eicosatrienoic acid	**C20:3n6**	0.00	0.00
Arachidonic acid	Eicosatetraenoic acid	**C20:4n6**	0.00	0.00
ETrA	Eicosatrienoic acid	**C20:3n3**	0.13	1.22
ETA	Eicosatetraenoic acid	**C20:4n3**	0.34	3.26
EPA	Eicosapentaenoic acid	**C20:5n3**	0.03	0.32
Behenic acid	Docosanoic acid	**C22:0**	0.14	1.36
Erucic acid	Docosenoic acid	**C22:1**	0.06	0.61
Docosadienoate	Docosadienoic acid	**C22:2n6**	0.00	0.00
Adrenic acid	Docosatetraenoic acid	**C22:4n6**	0.11	1.08
Osbond acid	Docosapentaenoic acid	**C22:5n6**	0.00	0.00
Clupanodonic acid	Docosapentaenoic acid	**C22:5n3**	0.00	0.00
DHA	Docosahexaenoic acid	**C22:6n3**	0.09	0.84
Lignoceric acid	Tetracosanoic acid	**C24:0**	0.00	0.00

Table S3: Criteria for nest quality scores

Criteria	Scoring details
Shredding	• 0 = nestlets are unshredded<br>• 0.5 = 1-25% shredded. 1 full nestlet square is left unshredded & 1 nestlet has been partially shredded but is still less than half-shredded.<br>• 1 = 26-50% shredded. One nestlet square remains unshredded. Other square is half- to fully-shredded.<br>• 1.5 = 51-75% - Both nestlets have been shredded/touched (at least a little). Sum of the chunks are approximately ¾ to ½ of one nestlet square<br>• 2 = 76-95% >1 pieces of nestlet material noticeable<br>○ Chunky texture<br>• 2.5 = >/= 96% - at most one small piece of unshredded material<br>○ Fluffy texture
Shape	• 0 = Scattered<br>○ Pieces of nest material are not all connected<br>○ Very dispersed within the cage<br>○ Scores of 0-1 for nest shredding<br>• 0.5 = Somewhat defined shape<br>○ Most of the material connects<br>○ Both of the following:<br>▪ > 2 edges are sticking out<br>▪ Very dispersed within the cage<br>• 1 = Mostly defined<br>○ All of the material connects<br>○ Mostly rounded shape<br>○ One of the following:<br>▪ > 2 edges are sticking out<br>▪ Very dispersed within the cage<br>• 1.5 = Defined shape<br>○ Round shape<br>○ Material is gathered *tightly* to ensure snug fit around the mouse
Location	• 0 = Front: touching front wall<br>• 0.5 = Middle<br>• 1 = Back: touching back wall
Nest walls	• 0 = < 25%<br>• 0.5 = 25-49%<br>• 1 = 50-75%<br>• 1.5 = > 75%

Table S4. Primer sequences

Gene	Forward Primer 5' to 3'	Reverse Primer 5' to 3'
RPLP0	ACTGGTCTAGGACCCGAGAAG	TCTGACCTCTGTTCCACCCT
IL-1β	AGTTGACGGACCCCAAAAG	GGACTACTCTCGTAGGTCGA
TNF-α	CATCTTCTCAAAATTCGAGTGACAA	CCCAACATGGAACAGATGAGGGT
TLR-4	AGAAAATGCCAGGATGATGC	CTGATCCATGCATTGGTAGGT
IL-10	CCTGGGTGAGAAGCTGAAGAC	CCTTGTAGACACCTTGGTCTTGG
16S	TCGTCGGCAGCGTCAGATGTGTATAAGAGACAGCCTACGGGNGGCWGCAG	GTCTCGTGGGCTCGGAGATGTGTATAAGAGACAGGACTACHVGGGTATCTAATCC

RPLP0: Ribosomal protein lateral stalk subunit P0, IL-1β: Interleukin 1 beta, TNF-α: Tumour necrosis factor alpha, TLR-4: Toll-like receptor 4, IL-10: Interleukin 10, 16S: 16S rRNA primer for amplicon PCR

Table S5. The relative abundance of the fecal microbiota pre-injection

Taxonomy	Relative abundance (% ±SEM)		
	BD	FS	FO
p__Bacteroidetes	51.019 ± 1.441	50.279 ± 1.428#	53.058 ± 1.002
f__S24-7;g__	35.188 ± 1.127	36.852 ± 0.873	36.844 ± 1.402
g__Bacteroides	9.919 ± 0.708	9.667 ± 0.851	9.642 ± 0.708
g__Parabacteroides	5.912 ± 0.417	3.76 ± 0.443*#	6.572 ± 0582
p__Firmicutes	29.591 ± 1.414	37.706 ± 1.244*#	26.644 ± 1.365
f__Ruminococcaceae; g__ (1)	3.087 ± 0.249	4.608 ± 0.483*#	2.917 ± 0.275
f__Ruminococcaceae; g__ (2)	2.583 ± 0.259	2.006 ± 0.163	2.474 ± 0.246
f__Lachnospiraceae; g__	5.264 ± 0.614	8.25 ± 0.62*#	3.923 ± 0.474
o__Clostridiales; f__	6.517 ± 0.485	8.370 ± 0.684*	6.705 ± 0.427
g__rc4-4	2.791 ± 0.349	1.362 ± 0.207*#	2.823± 0.189
g__Clostridium (f__Ruminococcaceae)	1.747 ± 0.206	2.372 ± 0.163*#	1.700 ± 0.197
g__Sporobacter	1.436 ± 0.29	1.291 ± 0.249	0.925 ± 0.136
g__Clostridium (f__Clostridiaceae)	1.013 ± 0.15	0.553 ± 0.111*	0.826 ± 0.158
g__Oscillospira	0.542 ± 0.1	0.372 ± 0.069	0.381 ± 0.060
g__Dorea	0.401 ± 0.044	0.185 ± 0.018*#	0.385 ± 0.043
g__Defluviittalea	0.380 ± 0.046	0.184 ± 0.023*#	0.299 ± 0.031
g__Lactobacillus	0.499 ± 0.177	0.405 ± 0.216*	0.372 ± 0.108
f__Bacillaceae;__	0.768 ± 0.201	3.335 ± 0.547*#	0.706 ± 0.206
g__Clostridium (f__Peptostreptococcaceae)	0.266 ± 0.065	0.03 ± 0.013*#	0.182 ± 0.061
o__Lactobacillales; f__	0.196 ± 0.017	0.156 ± 0.02#	0.216 ± 0.022
g__Clostridium (f__Lachnospiraceae)	1.266 ± 0.144	3.141 ± 0.198*#	1.022 ± 0.117
g__Ruminococcus (f__Lachnospiraceae)	0.114 ± 0.023	0.223 ± 0.034*#	0.119 ± 0.015
g__Allobaculum	0.193 ± 0.073	0.447 ± 0.081*#	0.213 ± 0.071
p__Verrucomicrobia	16.38 ± 1.576	9.019 ± 1.219*#	18.339 ± 1.239
g__Akkermansia	16.38 ± 1.576	9.019 ± 1.219*#	18.339 ± 1.239
p__Proteobacteria	1.831 ± 0.722	1.023 ± 0.391*#	0.885 ± 0.454
f__Enterobacteriaceae; g__	0.088 ± 0.02	0.204 ± 0.05*	0.157 ± 0.044
o__RF32 ;f__	1.743 ± 0.715	0.755 ± 0.391#	0.728 ± 0.451
p__Actinobacteria	0.074 ± 0.022	0.978 ± 0.313*#	0.264 ± 0.209
g__Bifidobacterium	0.018 ± 0.018	0.643 ± 0.305*	0.208 ± 0.207
f__Coriobacteriaceae	0.003 ± 0.002	0.202 ± 0.030*#	0.002 ± 0.002
p__Deferribacteres	0.478 ± 0.101	0.299 ± 0.092*#	0.307 ± 0.066
g__Mucispirillum	0.478 ± 0.101	0.299 ± 0.092*	0.307 ± 0.066
p__Tenericutes	0.608 ± 0.138	0.147 ± 0.065*#	0.44 ± 0.115
g__Anaeroplasma	0.608 ± 0.138	0.147 ± 0.065*#	0.44 ± 0.115
p__Cyanobacteria	0.019 ± 0.016	0.548 ± 0.275*#	0.064 ± 0.043
o__Streptophyta; f__	0.019 ± 0.016	0.335 ± 0.251*#	0.064 ± 0.043

Mean relative abundance (% ± SEM) of taxonomic groups in the fecal microbiota pre-injection at the Phylum level (grey), or the order, family, or genus level (white) with mean abundance greater than 0.2% in any group (n=24/group). * = significant difference compared to BD ($q<0.05$), # = significant difference from FO ($q<0.05$) as measured by Mann-Whitney tests with Two-stage step-up (Benjamini, Krieger, and Yekutieli) method for False Discovery Rate (FDR) which was set to 5%.

Table S6. The relative abundance of the fecal microbiota after SAL/LPS injection

Taxonomy	Relative abundance (% ± SEM)					
	BD-SAL	BD-LPS	FS-SAL	FS-LPS	FO-SAL	FO-LPS
p__Bacteroidetes	53.957 ± 1.76	51.488 ± 1.955	52.233 ± 1.727	43.421 ± 2.289	52.881 ± 1.381	50.558 ± 3.234
f__S24-7;g__	37.901 ± 1.641	31.208 ± 1.297	40.949 ± 1.65	29.822 ± 1.763	38.244 ± 2.141	26.647 ± 1.23
g__Bacteroides	9.699 ± 0.98	14.354 ± 1.375	8.372 ± 1.076	9.47 ± 0.933	8.414 ± 1.102	15.302 ± 1.848
g__Parabacteroides	6.357 ± 0.632	5.926 ± 0.537	2.912 ± 0.443	4.128 ± 0.454	6.223 ± 0.845	8.609 ± 0.975
p__Firmicutes	29.225 ± 1.679	22.983 ± 1.942	38.455 ± 1.851	32.032 ± 2.164	24.31 ± 1.656	24.517 ± 1.927
f__Ruminococcaceae;__	5.569 ± 0.881	5.362 ± 0.495	8.868 ± 0.757	8.034 ± 0.768	3.876 ± 0.599	6.688 ± 0.746
f__Lachnospiraceae;__	5.674 ± 0.447	2.537 ± 0.405	6.13 ± 0.459	5.656 ± 0.708	4.557 ± 0.477	2.287 ± 0.415
o__Clostridiales	7.606 ± 0.869	6.942 ± 0.644	8.377 ± 0.663	7.558 ± 0.853	6.167 ± 0.426	7.347 ± 0.682
g__rc4-4	2.334 ± 0.482	2.083 ± 0.312	1.075 ± 0.209	0.99 ± 0.199	2.928 ± 0.289	1.566 ± 0.282
g__Clostridium (f__Lachnospiraceae)	1.383 ± 0.207	0.753 ± 0.113	4.002 ± 0.396	3.075 ± 0.37	0.834 ± 0.097	0.56 ± 0.081
g__Sporobacter	1.659 ± 0.309	1.115 ± 0.385	1.779 ± 0.399	1.381 ± 0.24	0.994 ± 0.23	0.99 ± 0.387
g__Clostridium (f__Ruminococcaceae)	1.695 ± 0.183	1.42 ± 0.303	2.824 ± 0.3	2.273 ± 0.234	1.405 ± 0.169	1.723 ± 0.27
g__Lactobacillus	0.44 ± 0.247	0.28 ± 0.096	0.176 ± 0.047	0.116 ± 0.026	0.283 ± 0.116	0.314 ± 0.122
f__Bacillaceae;__	0.32 ± 0.108	0.107 ± 0.082	2.735 ± 0.564	0.522 ± 0.169	0.195 ± 0.095	0.068 ± 0.028
g__Dorea	0.384 ± 0.049	0.363 ± 0.043	0.274 ± 0.043	0.287 ± 0.055	0.348 ± 0.043	0.411 ± 0.069
g__Clostridium (f__Clostridiaceae)	0.224 ± 0.068	0.298 ± 0.094	0.305 ± 0.115	0.107 ± 0.049	0.765 ± 0.219	0.334 ± 0.117
g__Oscillospira	0.505 ± 0.058	0.405 ± 0.122	0.338 ± 0.071	0.31 ± 0.031	0.493 ± 0.068	0.539 ± 0.105
g__Defluviitalea	0.36 ± 0.036	0.203 ± 0.024	0.271 ± 0.046	0.249 ± 0.048	0.319 ± 0.045	0.387 ± 0.089
o__Lactobacillales	0.231 ± 0.046	0.127 ± 0.026	0.219 ± 0.024	0.091 ± 0.018	0.211 ± 0.017	0.106 ± 0.012
g__Allobaculum	0.096 ± 0.057	0.027 ± 0.016	0.396 ± 0.91	0.14 ± 0.053	0.094 ± 0.062	0.089 ± 0.063
g__Clostridium (f__Erysipelotrichaceae)	0.102 ± 0.035	0.21 ± 0.072	0.072 ± 0.025	0.374 ± 0.144	0.084 ± 0.022	0.338 ± 0.065
g__Papillibacter	0.086 ± 0.048	0.116 ± 0.056	0.104 ± 0.026	0.109 ± 0.03	0.101 ± 0.041	0.21 ± 0.044
p__Verrucomicrobia	13.974 ± 2.587	21.225 ± 1.745	7.368 ± 1.466	16.579 ± 2.01	19.603 ± 1.414	21.164 ± 2.663
g__Akkermansia	13.974 ± 2.587	21.225 ± 1.745	7.368 ± 1.466	16.579 ± 2.01	19.603 ± 1.414	21.164 ± 2.663
p__Proteobacteria	1.815 ± 1.317	3.313 ± 1.868	0.743 ± 0.242	7.113 ± 2.152	0.782 ± 0.282	3.191 ± 1.243

f__Enterobacteriaceae	0.4 ± 0.11	3.148 ± 1.879	0.603 ± 0.195	7.071 ± 2.153	0.429 ± 0.136	3.124 ± 1.256
o__RF32	1.415 ± 1.274	0.165 ± 0.157	0.039 ± 0.031	0.012 ± 0.008	0.353 ± 0.248	0.067 ± 0.065
p__Actinobacteria	0.066 ± 0.014	0.042 ± 0.012	0.817 ± 0.370	0.198 ± 0.043	0.068 ± 0.018	0.123 ± 0.03
g__Bifidobacterium	0 ± 0	0 ± 0	0.545 ± 0.363	0.043 ± 0.038	0.001 ± 0.001	0 ± 0
p__Deferribacteres	0.475 ± 0.154	0.613 ± 0.127	0.21 ± 0.048	0.475 ± 0.178	0.149 ± 0.025	0.382 ± 0.099
g__Mucispirillum	0.475 ± 0.154	0.613 ± 0.127	0.21 ± 0.048	0.475 ± 0.178	0.149 ± 0.025	0.382 ± 0.099
p__Tenericutes	0.395 ± 0.147	0.326 ± 0.193	0.058 ± 0.03	0.073 ± 0.06	0.269 ± 0.09	0.062 ± 0.03
g__Anaeroplasma	0.395 ± 0.147	0.326 ± 0.193	0.058 ± 0.03	0.073 ± 0.06	0.269 ± 0.09	0.062 ± 0.03
p__Cyanobacteria	0.033 ± 0.031	0.126 ± 0.126	0.938 ± 0.535	0.403 ± 0.087	0.066 ± 0.065	0.2 ± 0.199
o__Streptophyta	0 ± 0	0 ± 0	0.310 ± 0.075	0.108 ± 0.035	0 ± 0	0 ± 0
o__YS2	0.126 ± 0.126	0.010 ± 0.009	0.093 ± 0.076	0 ± 0	0.200 ± 0.199	0.003 ± 0.003

Mean relative abundance (% ± SEM) of taxonomic groups in the fecal microbiota pre and post LPS injection at the Phylum level (grey), or the class, order, family, or genus level (white) with mean abundance greater than 0.2% in any group (n=12/group).

Table S7: Clades reduced by an LPS challenge (corresponds to Figure 8d)

Diet groups	Number of Similarities	Specific clades
BD FO FS	5	S24-7 Lachnospiraceae Lactobacillales Clostridium Clostridia
BD FS	1	Firmicutes
BD	2	Defluviitalea Ruminococcaceae
FS	18	4C0d_2 Paraeggerthella Bacteroidetes Coriobacteriaceae Streptophyta Cyanobacteria Bacteroidia YS2 Coriobacteriales Actinobacteria Bacteroidales Bacillales Allobaculum Chloroplast Clostridiaceae Coriobacteriia Bacilli Bacillaceae
FO	2	rc4-4 Peptococcaceae

Table S8: Clades increased by an LPS challenge (corresponds to Figure 8e)

Diet groups	Number of Similarities	Specific clades
BD FS	8	Gammaproetobacteria Verrucomicrobiaceae Verrucomicrobiales Enterobacteriales Enterobacteriaceae Akkermansia Verrucomicrobioae Verrucomicrobia
BD FO	2	Bacteroides Bacteroidaceae
FO FS	1	Clostridium
FS	3	Proteobacteria Ruminococcus Ruminococcaceae (2)
FO	10	Erysipelotrichaceae Erysipelotrichi Deferribacterales Erysipelotrichales Parabacteroides Papillibacter Deferribacteres Porphyromonadaceae Mucispirillum Deferribacteraceae
